# Atomic Layer Deposition—A Versatile Toolbox for Designing/Engineering Electrodes for Advanced Supercapacitors

**DOI:** 10.1002/advs.202303055

**Published:** 2023-11-08

**Authors:** Mohd Zahid Ansari, Iftikhar Hussain, Debananda Mohapatra, Sajid Ali Ansari, Reza Rahighi, Dip K Nandi, Wooseok Song, Soo‐Hyun Kim

**Affiliations:** ^1^ School of Materials Science and Engineering Yeungnam University 280 Daehak‐Ro Gyeongsan Gyeongbuk 38541 Republic of Korea; ^2^ Department of Mechanical Engineering City University of Hong Kong 83 Tat Chee Avenue Kowoon Hong Kong; ^3^ Graduate School of Semiconductor Materials and Devices Engineering Ulsan National Institute of Science & Technology (UNIST) 50 UNIST‐gil Ulju‐gun Ulsan 44919 Republic of Korea; ^4^ Department of Physics College of Science King Faisal University P.O. Box 400 Hofuf Al‐Ahsa 31982 Saudi Arabia; ^5^ SKKU Advanced Institute of Nano‐Technology (SAINT) Sungkyunkwan University 2066 Seobu‐ro, Jangan‐gu Suwon Gyeonggi‐do 16419 Republic of Korea; ^6^ Plessey Semiconductors Ltd Tamerton Road Roborough Plymouth Devon PL6 7BQ UK; ^7^ Thin Film Materials Research Center Korea Research Institute of Chemical Technology Daejeon 34114 Republic of Korea; ^8^ Department of Materials Science and Engineering Ulsan National Institute of Science & Technology (UNIST) 50 UNIST‐gil Ulju‐gun Ulsan 44919 Republic of Korea

**Keywords:** atomic layer deposition (ALD), thin films, electrode materials, electrode architecture designs, performance optimization

## Abstract

Atomic layer deposition (ALD) has become the most widely used thin‐film deposition technique in various fields due to its unique advantages, such as self‐terminating growth, precise thickness control, and excellent deposition quality. In the energy storage domain, ALD has shown great potential for supercapacitors (SCs) by enabling the construction and surface engineering of novel electrode materials. This review aims to present a comprehensive outlook on the development, achievements, and design of advanced electrodes involving the application of ALD for realizing high‐performance SCs to date, as organized in several sections of this paper. Specifically, this review focuses on understanding the influence of ALD parameters on the electrochemical performance and discusses the ALD of nanostructured electrochemically active electrode materials on various templates for SCs.

It examines the influence of ALD parameters on electrochemical performance and highlights ALD's role in passivating electrodes and creating 3D nanoarchitectures. The relationship between synthesis procedures and SC properties is analyzed to guide future research in preparing materials for various applications. Finally, it is concluded by suggesting the directions and scope of future research and development to further leverage the unique advantages of ALD for fabricating new materials and harness the unexplored opportunities in the fabrication of advanced‐generation SCs.

## Graphical abstract

This paper offers a thorough review of atomic layer deposition (ALD) and its applications in supercapacitor electrode development. It scrutinizes the impact of ALD parameters on electrochemical performance, explores its utility in creating 3D nanoarchitectures, and examines the correlation between synthesis techniques and supercapacitor properties. It concludes by outlining potential future research directions to further exploit ALD's unique benefits in the fabrication of advanced supercapacitors.

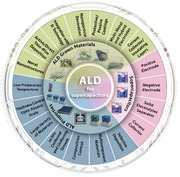

## 1 Introduction

**Figure 1:**
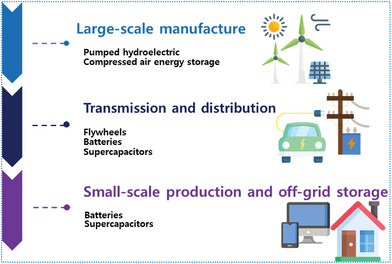
Technologies for energy production are utilized at a variety of scales in the power source.

The worldwide consumption of already declining reserves of fossilized fuels has necessitated the replacement of gasoline‐based vehicles with novel devices driven on alternate sources of energy.^[^ ^1^, ^2^, ^3^ ^]^ Currently, we are witnessing a societal‐level shift in the energy paradigm as the energy demand increases tremendously.^[^ ^1^, ^4^ ^]^ The unbridled exploitation of fossil fuels in the past has arguably depleted their future availability. One of the major concerns of the twenty‐first century is to identify and develop appropriate and effective energy‐storage systems to fully realize the potential of diverse renewable energy systems (**Figure** **1**).^[^ ^2^ ^]^ Electrochemical energy storage with high energy/power deliveries, prolonged cyclic lifespan, and high efficiency are imminently required for applications in wearable and portable electronic devices, electric and hybrid electric vehicles, and grid energy storage.^[^ ^5^, ^6^, ^7^, ^8^, ^9^ ^]^ Among various unconventional electric‐power devices, rechargeable batteries and electrochemical supercapacitors are two foremost electrochemical systems that are frequently employed in electric powered vehicles as well as compact and wearable consumer electronics.^[^ ^10^, ^11^, ^12^, ^13^, ^14^, ^15^, ^16^, ^17^, ^18^, ^19^, ^20^, ^21^, ^22^, ^23^ ^]^

**Figure 2:**
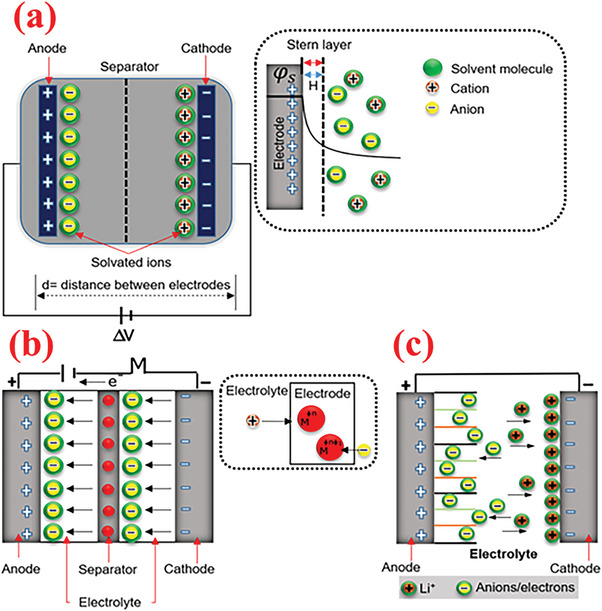
Graphic representation of supercapacitors based on various charge‐storage mechanisms of a) EDLCs, b) PCs, and c) hybrid capacitors. Reproduced with permission.^[^ ^24^ ^]^ Copyright 2020, Elsevier.

Supercapacitors (SCs) are at the forefront of the continuous quest for energy sustainability owing to their superior properties such as high specific capacitance, fast charge‐storage capability (low charge/discharge (CD) period of 1–10 s), minimal maintenance requirements, zero memory effects, high safety, and excellent cycling properties.^[^ ^24^, ^25^, ^26^, ^27^, ^28^, ^29^, ^30^, ^31^, ^32^, ^33^, ^34^, ^35^, ^37^, ^38^, ^39^, ^40^, ^41^ ^]^ Moreover, SCs can deliver high power density at higher energy densities, enabling them to overcome the power–energy imbalance between capacitors (high power capability^[^ ^13^ ^]^) and batteries and fuel cells (high energy density) compared to other known storage technologies. Unlike secondary batteries, SCs exhibit higher power densities (500–10,000 W kg^–1^ for SCs vs < 1000 W kg^−1^ for batteries), shorter CD period (1–10 s for SCs vs 0.5–5 h for batteries), superior lifespan (>500 000 h for SCs vs 500–1000 h for batteries) and improved operational safety.^[^ ^3^, ^5^, ^42^, ^43^, ^44^, ^45^ ^]^ Nevertheless, the lower energy density of SCs (1–10 Wh kg^−1^) than batteries (10–100 Wh kg^–1^) is a major limitation to their commercial viability.^[^ ^15^, ^45^, ^46^ ^]^ Among the potential strategies of circumventing the problem of inferior energy delivery of SCs, the development of novel high‐performance electrode materials forms an effective and facile approach that has been extensively pursued. In a capacitor, the charge is electrostatically stored in the metal plates and an electric field emerges across the dielectric medium.^[^ ^34^, ^47^, ^48^, ^49^, ^50^, ^51^, ^52^ ^]^ The SCs store charges via multiple mechanisms–reversible adsorption/desorption of ions between the surface of the active materials/electrodes and electrolytes, and swift and changeable Faradaic process at the surface of electrodes during the charge and discharge processes.^[^ ^3^, ^53^, ^54^, ^55^, ^56^, ^57^, ^58^, ^59^, ^60^, ^61^, ^62^, ^63^, ^64^, ^65^, ^66^, ^67^ ^]^ Depending upon the mechanism involved in charge storage, the SCs can be classified into three classes: electrical double layer capacitor (EDLC), pseudocapacitors (PCs), and hybrid SCs (**Figure** **2**).^[^ ^24^, ^68^, ^69^, ^70^, ^71^, ^72^ ^]^

Although energy and power are frequently used interchangeably, each refers to distinct characteristics of the device. In principle, energy represents the amount of work done, whereas power denotes the rate of energy used in completing this work.^[^ ^64^, ^74^, ^75^, ^76^, ^77^, ^78^, ^79^, ^80^, ^81^, ^82^, ^83^, ^84^, ^85^, ^86^, ^87^, ^88^, ^89^ ^]^ Thus, power can be defined as a measure of the energy consumed per unit of time. Thus, both energy and power are correlated by the expression:

Power=Energy/Time.

**Figure 3:**
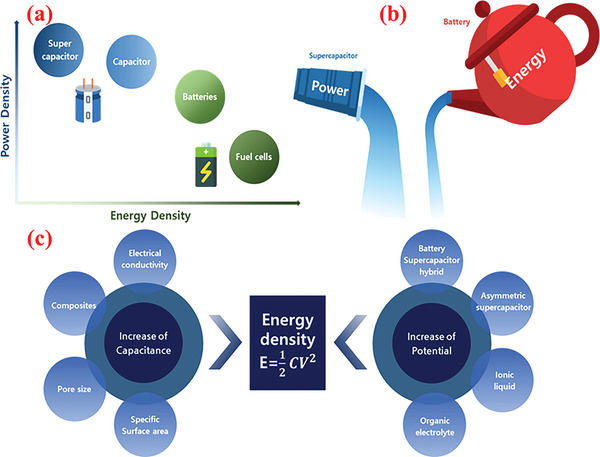
a) Ragone plot of the power–energy density range for various standard energy storage and conversion devices. b) Schematic illustration of power density vs. energy density. c) Schematic of various routes to increase the energy density of supercapacitors.

A Ragone plot of energy and power density of significant energy storage/conversion devices is illustrated in **Figure** **3**a.

Energy and power contribute as the most vital characteristics of energy storage devices. As observed from the Ragone plot, conventional capacitors, SCs, and batteries exhibit various levels of energy and power density across the spectrum.^[^ ^47^, ^73^, ^74^, ^75^, ^76^, ^77^, ^78^, ^79^, ^80^, ^81^ ^]^ Although energy density denotes the amount of energy contained in a device, the power density governs the delivery or discharge speed of this energy.^[^ ^99^, ^100^, ^101^, ^102^, ^103^, ^104^, ^105^ ^]^ Capacitors provide a large amount of power but can store substantially less charge (energy density), similar to a glass of water depicted in Figure 3b. Although a battery stores a significantly greater amount of charge, it charges and discharges slowly, analogous to a large water bottle (Figure 3b). As discussed, SCs fundamentally suffer from low energy densities and their performance is evaluated by the essential factors of energy (*E*) and power (*P*) densities. The equations for calculating *E* and *P* are expressed in Figure 3c. As the energy density of a capacitor relies directly on the specific capacitance of the materials as well as the square of the cell voltage (*V*), the focus is to improve the capacitance or potential window or both of these parameters. In principle, these parameters predominantly rely on the nature and character of the electrode materials used in SCs. Despite the facile production of the individual constituents (e.g., electrode material, electrolyte) of SCs, the compatibility between the pore structure and dimension of the electrode/active materials and the size of electrolyte ion is crucial for achieving a synergistic effect and improving the electrochemistry of the overall device.^[^ ^82^, ^83^, ^84^, ^85^, ^86^, ^87^, ^88^, ^89^ ^]^

The synthesis process allows for direct control of the structure and properties of electrode materials. By employing various strategies such as developing nanostructured active materials, optimizing constituents, and creating novel cell configurations, the challenges faced by SCs have been addressed.^[^ ^46^, ^47^, ^64^, ^90^, ^91^, ^92^, ^93^, ^94^, ^95^, ^96^, ^97^, ^98^, ^99^, ^100^, ^101^, ^102^, ^103^, ^104^, ^105^, ^106^, ^107^, ^108^ ^]^ One significant challenge in fabricating nanostructured materials is manipulating materials with both structural and chemical differences at the nanometer scale. Combining a single material with ideal support characteristics, such as a large specific surface area and superior physicochemical properties, with another material offering unique properties, poses a frequent difficulty.^[^ ^1^, ^99^, ^109^, ^110^, ^111^, ^112^, ^113^, ^114^, ^115^, ^116^, ^117^, ^118^ ^]^ Among the potential solutions, the surface coating/decoration of active materials is a widely reported technique of promoting the electrochemistry of SCs. Depending on the properties of the electrodes, several kinds of coating materials have been demonstrated thus far.^[^ ^119^, ^120^, ^121^ ^]^ The coating technique employed is also well‐established to influence the performance parameters of SCs. Consequently, a wide array of synthesis procedures encompassing mechanical mixing, hydrolysis‐precipitation, and sol–gel has been developed.^[^ ^1^, ^47^, ^49^ ^]^ However, these techniques have purity issues, require high temperature, and lack synthesis control and uniformity control over the thickness of deposited layer. Thus, a method that can synthesize and manipulate the surface with good thickness control and nanoscale uniformity coating (or even angstrom level) should be developed to maximize the benefits of nanomaterials and limit their adverse impacts. In this perspective, ALD boasts of the most dominant synthesis technique of preparing novel materials to fulfill the technological needs of future‐generation sustainable energy. Propelled by the advantages of excellent conformity, superior thickness, composition control, as well as the ease of atomic‐scale manipulation, ALD is a promising deposition strategy for studying, designing, and fabricating required nanomaterials.^[^ ^119^, ^120^, ^121^ ^]^ Additionally, an overwhelming variety of materials and structures, e.g., wafers, nanoparticles, nanowires, nanotubes, soft materials, and biomaterials, have been successfully coated via the ALD process.^[^ ^122^, ^123^, ^124^, ^125^, ^126^, ^127^, ^128^, ^129^, ^130^, ^131^ ^]^ Furthermore, owing to their improved properties, ALD‐deposited materials have been extensively applied in various fields such as batteries, SCs, catalysis, and sensors.^[^ ^122^, ^131^, ^132^, ^133^ ^]^

## 2 Fundamentals of Atomic Layer Deposition (ALD)

In principle, ALD provides a gaseous and chemical route for depositing pristine and uniform thin films. Recently, it has become the focus of increasing attention as a state‐of‐the‐art thin‐film deposition tool across a wide range of modern technological applications. Owing to the controlled growth process, the obtained films are highly conformal. Although ALD is a potential derivative of the well‐established chemical vapor deposition (CVD) technique, these two processes clearly differ based on the introduction of the gas‐phase precursors. In contrast to CVD, the precursors in ALD are introduced in an alternate manner. The term “ALD” originates from “atomic layer epitaxy (ALE),” a technique employed for depositing ZnS over flat display panels.^[^ ^134^, ^135^, ^136^ ^]^ The unrelenting demand for continuous and pinhole‐free films further propelled the developments in semiconducting device technologies, which eventually yielded the advancements in atomic deposition techniques. In the mid‐1970s, Finnish researchers Suntola et al. patented the ALE technique for the first time. Since this patent, the modern techniques of ALD have progressed considerably to emerge as a well‐established technique in scientific and industrial applications.^[^ ^137^, ^138^, ^139^, ^140^ ^]^ The commercial significance of ALD was first realized during the early 2000s as a consequence of its adoption by the semiconductor industry to manufacture high‐performing complementary metal–oxide semiconductor (CMOS) transistors.^[^ ^123^, ^127^, ^128^, ^141^, ^142^, ^143^ ^]^ Since 2007, both Intel and IBM have been using ALD to produce highly dielectric layers for CMOS integration.^[^ ^129^, ^136^, ^139^, ^140^, ^144^, ^145^ ^]^ In contrast to other deposition methods, ALD is a gas‐phase technique that deposits the desired material by involving two gas–solid half‐reactions. The advancements in nanotechnology offer unique opportunities for ALD‐deposited materials in novel application areas.

### 2.1 ALD Principle

**Figure 4:**
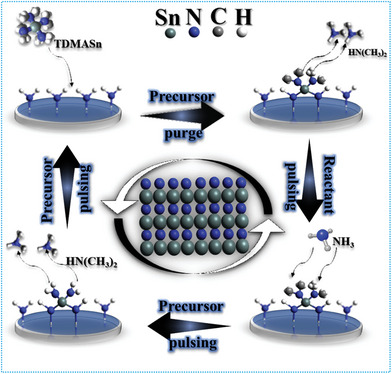
Schematic of ALD of tin nitride (SnN_x_) process using TDMASn and NH_3_ precursors as an example.

In contrast to conventional techniques such as CVD and PVD that depend on the primary sources of the reactants, the film growth behavior in ALD is controlled through surface‐limited chemisorption that governs the half reactions of the self‐limited gas–solid surface.^[^ ^144^, ^146^, ^147^, ^148^, ^149^, ^150^, ^151^, ^152^, ^153^, ^154^, ^155^ ^]^ To exhibit the fundamental mechanisms of the ALD processes, an ALD progression for the binary compound SnN_x_ is visually demonstrated in **Figure** **4**. As such, binary ALDs can be produced with two precursors. As displayed in Figure 4, an ALD procedure of SnN_x_ (ALD–SnN_x_) utilizes tetrakis(dimethylamino) tin (TDMASn, Sn(NMe_2_)_4_) and ammonia (NH_3_) as its precursors.^[^ ^156^ ^]^ Consequently, these characteristics enable the individual reaction of each precursor and reactant over the surface of the heated substrate, which creates new surface layers for the subsequent reactions. The layer growth ceases entirely after the chemical reaction is complete, the substrate surface is covered, and the reactor is thoroughly cleansed or purged before introducing the subsequent precursor on the substrate.

The ALD of SnN_x_ involves the following half‐reactions^[^ ^156^ ^]^

(1) $$2\mathrm{NH}*+\mathrm{Sn}{\left(N{\left({\mathrm{CH}}_{3}\right)}_{2}\right)}_{4}\to N_{2}\mathrm{Sn}{\left(N{\left({\mathrm{CH}}_{3}\right)}_{2}\right)}_{2}*+2\mathrm{HN}{\left({\mathrm{CH}}_{3}\right)}_{2}$$

(2) $$N_{2}\mathrm{Sn}{\left(N{\left({\mathrm{CH}}_{3}\right)}_{2}\right)}_{2}*+NH_{3}\to N_{2}{\mathrm{SnNH}}_{2}*+2\mathrm{HN}{\left({\mathrm{CH}}_{3}\right)}_{2}+N_{2}$$

where the species transferred or anchored on a substrate via chemisorption are marked with an asterisk (*), and (g) denotes the gaseous nature of the species. Reactions (1) and (2) are both transamination exchange reactions in which the NH_x_* surface species is replaced by the Sn(N(CH_3_)_2_)*_y_* * surface species and vice versa.

### 2.2 Growth Properties

#### 2.2.1 Linearity, Saturation, and ALD Window

A significant characteristic of typical ALD growth is that the thickness of the deposited material increases linearly with the number of growth cycles. The amount of material added or the equivalent increase in thickness per ALD cycle is typically defined as the “growth per cycle” (GPC).^[^ ^124^, ^128^, ^143^, ^157^, ^158^ ^]^

**Figure 5:**
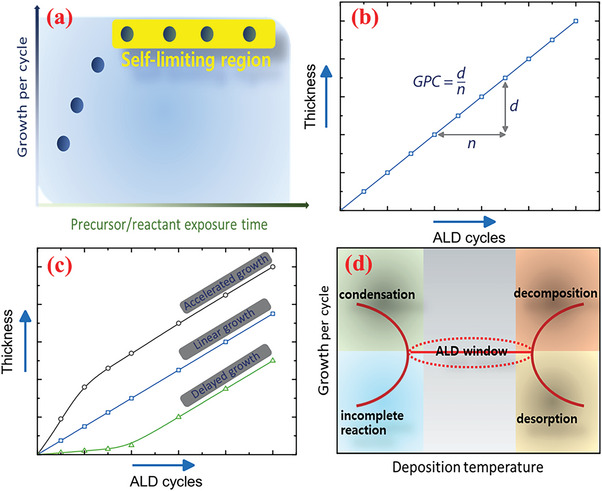
Schematic of a) GPC saturation for both precursors behavior of ideal ALD process. b) ALD process with multiple ALD cycles, displaying linear growth characteristics of a typical ALD.^[^ ^141^ ^]^ c) Certain growth types probable in ALD as functions of ALD cycles.^[^ ^141^ ^]^ d) ALD process as function of deposition temperature.

The growth mechanism predicts that for adequately high concentrations of both precursors, the GPC should become saturated and the deposition reaction must continue with the reactant concentration in the saturation region (precursor *vs*. GPC scenario), as depicted in **Figure** **5**a. In this regard, a saturation deficit in the growth processes indicates a tendency toward the CVD type of growth.^[^ ^142^, ^143^ ^]^ Thus, GPC should be independent of the number of cycles, and ideally, remain constant over all ALD cycles (linearity or cycle *vs*. thickness) (Figure 5b).^[^ ^141^ ^]^ Therefore, the thickness achieved in a specific number of cycles can be accurately predicted.^[^ ^141^ ^]^ Generally, if the deposited material differs from the substrate material, deviations from the ideal growth behavior are observed for the first few cycles (≈10). In this case, the nucleation of the film over the substrate surface is delayed, resulting in lower achievable values of GPC (Figure 5c).^[^ ^141^ ^]^ Upon attaining complete surface coverage, the GPC attains its final value. As the ALD growth process is unrestricted by the mass transport, the GPC values should not be affected by the growth temperatures within limits. The temperature range in which the growth process satisfies the conditions of the self‐limited reaction is referred to as the “ALD process window” (Figure 5d). Beyond this temperature window, various physicochemical activities can interrupt the deposition reaction.^[^ ^123^, ^141^, ^143^ ^]^ Thus, to realize the benefits of ALD, the process must be operated within a suitable temperature window.

#### 2.2.2 Low Temperature

A major reason for the growing popularity of the ALD process is its potential to grow ultrahigh‐quality thin‐films at relatively lower reaction temperatures. As the growth process involves a complete reaction between the adsorbed precursor species and reactants, the resultant films are relatively purer than their CVD counterparts even at lower reaction temperatures.^[^ ^123^, ^142^, ^143^ ^]^ Nevertheless, the adsorption of precursors and the surface reaction between the reactants in ALD require a thermal activation that is typically provided via substrate heating. Furthermore, ALD enables the deposition of highly pure films of certain materials even at growth temperatures below 100 °C.^[^ ^154^ ^]^ Low temperatures may condense the reactants on the surface or cause thermal energy deficiency in surface reactions attempting to cover the entire surface. Thus, the surface species may readily decompose under exceedingly high temperatures. Therefore, ALD deposition can occur inside their corresponding ALD windows. The ALD method has been implemented for synthesizing various kinds of materials such as tellurides, sulfides, oxides, selenides, and nitrides for various bespoke applications.^[^ ^123^, ^159^, ^160^, ^161^, ^162^, ^163^, ^164^ ^]^

#### 2.2.3 Uniformity and Conformality

As a self‐terminating process, ALD enables the growth of high‐quality, uniform, and conformal thin‐films even in structures with ultrahigh depth‐to‐width ratios.^[^ ^152^ ^]^ As ALD deposits uniform layers of active materials, it permits the construction of novel nanostructures with superior properties compared to those fabricated by alternative techniques such as PVD or CVD.^[^ ^165^ ^]^ The excellent conformity of the deposited films is one of the most significant advantages of ALD. The conformality of ALD‐grown films is generally established by depositing materials in a deep micron‐sized trench (width: 1 µm) and examining the structure via cross‐sectional electron microscopy. A uniform film demonstrates a constant composition and thickness (including other desired properties) at each point along the surface of a planar substrate, for instance, along a 300‐mm‐wide wafer. In contrast, a conformal film contains an identical thickness inside the 3D structures, i.e., an “around‐the‐corner” uniformity.^[^ ^173^ ^]^ However, to achieve highly conformal films inside/outside the multifaceted constructions, numerous aspects should be considered, such as sticking probability, surface diffusion, and precursor flux. Thus, the reactants should permit chemisorption over the entire substrate surface.^[^ ^173^ ^]^

#### 2.2.4 Atomic‐Scale and Stoichiometric Deposition

As discussed, the self‐limiting mode of ALD facilitates layer‐by‐layer growth with controllable thickness and composition at the Angstrom‐scale. The high conformity of the deposited films accounts for the most significant merit of ALD. The inherent self‐terminating nature of ALD by alternate exposure provides excellent control over the composition and thickness at the atomic scale.^[^ ^129^, ^166^, ^167^, ^168^, ^169^ ^]^ Therefore, ALD is an ideal method of producing atomic‐scale thin‐films as well as layered nanostructures. Owing to these merits, ALD is a preferred film deposition technique over PVD, CVD, and other solution‐processing approaches. The deposition rate in an ALD process is governed by the used precursors, reaction temperatures, as well as the target substrate, and its typical values range within a few angstroms (generally < 2 Å per cycle).^[^ ^123^, ^134^, ^135^, ^145^, ^146^, ^157^, ^170^, ^171^, ^172^, ^173^, ^174^ ^]^ In addition to the control of deposition rates, ALD offers excellent control over the stoichiometry of the deposited material. In this perspective, the stoichiometry of ALD‐grown layers almost corresponds to the theoretical predictions; however, the reaction temperatures along with the precursor materials can affect the resulting films. For instance, several physical characteristics of the ALD‐grown SnS_x_ films can be tuned by employing TDMASn and hydrogen sulfide (H_2_S) as precursors to Sn^4+^ and sulfur.^[^ ^175^ ^]^ Overall, the ALD technique can readily alter the composition of the resultant tin sulfides (SnS and SnS_2_) by adjusting the deposition temperatures. Therefore, the control over the deposited layer compositions and growth rates considerably depends on the appropriate selection of the ALD parameters.

#### 2.2.5 Pros and Cons of ALD

ALD is a sophisticated thin‐film deposition technique that offers precise control at the atomic level, resulting in highly uniform and conformal thin films. Its primary advantage lies in its exceptional thickness and composition control, allowing for the fabrication of nanostructures with high aspect ratios and precise atomic layer thickness. It is highly repeatable and can deposit a variety of materials Moreover, ALD can be performed at relatively low temperatures, making it compatible with various substrate materials. On the flip side, the disadvantages of ALD primarily revolve around its time‐consuming nature and high operating cost. The layer‐by‐layer deposition process can be slow, especially for thicker films, and the precursor materials used in the process can be expensive (**Table** **1**). In the ALD process, about 60% of the precursor material is typically not utilized and is thus wasted, indicating a considerable loss of energy and the associated labor. Experimental evidence shows that the material utilization efficiency in the ALD process is somewhat low, with only ≈50% of TMA effectively deposited on the wafers. ^[^ ^158^, ^176^, ^177^, ^178^ ^]^ Furthermore, despite its ability to deposit various materials, some materials can be challenging due to the lack of appropriate precursors or the complexity of the required reactions.

**Table 1:** Possible Pros and Cons of ALD technique.

Pros	Cons
Superior Film Quality: Variety of materials, Precise control over the film's thickness, outstanding consistency, high density of the film, capability to generate either amorphous or crystalline films, competency in creating ultra‐slim films	Low growth rate
Very uniform and conformal deposition on any complex high‐aspect ratio structures	The lengthy duration required for the chemical reactions
Management of Complex Substrates: Gentle deposition procedure suitable for sensitive substrates, reduced temperature and stress, exceptional adhesion	Questions on the economic feasibility due to higher costs
Processing at Lower Temperatures	Significant material wastage rate
Control over Stoichiometry	Excessive energy consumption
Self‐assembled characteristic of the ALD mechanism	Risk of nanoparticle emissions
Capability to fabricate multilayer architectures	The intense nature of the ALD procedure

Recently, the research and development of ALD has garnered significant attention as a state‐of‐the‐art thin‐film deposition tool, finding widespread application across various modern technological domains. Researchers have been focusing on using ALD to synthesize and modulate 2D transition metal chalcogenides, showcasing their potential for diverse applications. ^[^ ^179^ ^]^ ALD's ability to precisely control material deposition at the atomic level has become indispensable for producing high‐quality and uniform thin films of these materials, making them essential for next‐generation devices. Additionally, ALD has emerged as a powerful method for fabricating metal oxides and chalcogenides, particularly for high‐performance transistors. ^[^ ^180^ ^]^ The technique's precise control over film thickness and composition enables researchers to engineer materials with custom electronic properties, enhancing device performance and enabling novel circuit designs. The advancements in ALD detailed in the research articles have solidified its position as a cutting‐edge thin‐film deposition tool, promising to revolutionize various technological fields.

### 2.3 ALD Applications for SCs

Owing to the advantageous properties of high‐power delivery, swift CD rates, exceptional cyclability and economic operation, electrochemical SCs have garnered immense consideration as a potential candidate for the future development of high‐performance energy‐storage systems. Generally, high‐capacity electrodes require a large specific surface area along with high electrical conductivity to facilitate the double‐layer formation and/or support redox kinetics. ^[^ ^3^, ^24^, ^75^ ^]^ Therefore, suitable production techniques are imminently required to produce SC electrode materials with desirable geometry, suitable charge transport behavior, controlled thickness, and surface chemistry. By leveraging its unique processing conductions, ALD has emerged as the most dominant technique for the development of advanced‐generation SC electrode materials with superior macro‐/nanoscale structures and surface characteristics. The advantages of ALD—–superior thickness control of the deposited layer, uniform deposition over a large surface area, conformity of deposition over intricate features, and ease of fabricating composite nanostructures—–render it particularly suitable for application in energy storage devices. Over the past decades, numerous review articles have extensively discussed the studies on ALD applications, especially those pertaining to energy storage and conversion.^[^ ^179^, ^181^, ^182^, ^183^, ^184^, ^185^, ^186^, ^187^, ^188^, ^189^, ^190^, ^191^ ^]^ Several remarkable reviews primarily concentrated on ALD‐based electrode materials for rechargeable batteries.^[^ ^192^, ^193^, ^194^, ^195^, ^196^, ^197^, ^198^, ^199^, ^200^, ^201^, ^202^, ^203^, ^204^, ^205^ ^]^ Among these, several notable reviews have been primarily dedicated to exploring ALD‐based electrode materials in the context of rechargeable batteries. Such investigations are crucial in our pursuit of improved energy storage solutions, and these comprehensive analyses have significantly advanced our understanding in this area. However, upon an extensive review of the existing literature, it appears that no study has yet assembled the most recent research on the use of ALD in fabricating materials specifically for SCs. The applications of ALD have been widely investigated since the start of the previous decade, yielding a substantial body of research output. Yet, a systematic and comprehensive review article detailing all aspects of this research—–including the varied deposition approaches employed, the unique properties exhibited by the materials produced, and the diverse range of applications these materials enable—–seems to be missing from the current literature. Given this situation, the need for an up‐to‐date and comprehensive review article becomes clear. Such a review would serve not only to compile and synthesize the latest findings in this field but also to provide a coherent and accessible reference point for both new researchers entering this field and established expert looking for a concise summary of recent advancements.

The present review reports the major applications of ALD, considering that the ALD process is admirably recognized for producing a wide range of materials applicable in various purposes. Among these published literatures, fewer reviews focused on SC in comparison to other applications of ALD‐based materials because ALD gained its prominence as an efficient tool for fabricating electrode materials only in 2010. Thus, the current review aims to comprehensively explore the applicability of ALD in SCs and its uses in flexible/chip‐based/wearable devices. However, an organized and comprehensive review and summary of applying the ALD‐based materials as SCs electrode is reasonably limited, particularly for the research development of the assembly of 3D/complex nano‐ and micro‐based architectures and free‐standing electrodes. Therefore, a current summary is required to examine the rapid development of innovative ALD‐grown materials in the SCs sector. Accordingly, we attempt to outline and focus on the progress of the most current advancements of ALD‐grown SCs electrodes, including storage mechanisms, various preparation procedures, and the corresponding electrochemical performances. Foremost, this paper provides background information on SCs and the ALD process, describing the various types of charge‐storage and ALD growth mechanisms to aid the understanding of the current assessment. Specifically, we focused on the ALD electrode to emphasize the applicability of ALD toward SCs. This review provides its readers with information on the real and ideal application of this technique in the field of SCs and the effective synthesis procedures typically adopted for ALD.

## 3 Status of ALD in SCs

The development of nanoscale or nanostructured materials is a vital research area in this perspective because of the unique enhancements of material properties at these dimensions vis‐a‐vis their bulk counterparts.^[^ ^8^, ^48^, ^49^ ^]^ Therefore, efficient preparation methods are imminently required for constructing SC electrodes exhibiting the desired geometry, thickness, and surface kinetics for effective charge‐transport characteristics. Compared to conventional thin‐film deposition processes such as PLD and CVD, ALD is emerging as a state‐of‐the‐art technique for depositing high‐quality, stoichiometric thin‐films of diverse materials. The novel processing conditions of ALD are specifically suited for developing SCs, where the device performance is governed by the surface chemistry and macro‐/nanoscale structures. Consequently, ALD can be used to address the following challenges regarding SC development: i) synthesis of high‐surface‐area nanosized current collectors; ii) deposition of nanostructured active materials in desired shape, thickness, etc.; iii) conformal coating of active material with protective layers and/or other desirable materials to enhance overall device performance; iv) fabrication of thin films of active materials, separators, electrolyte, current collectors, etc., which are essential for device miniaturization; v) finally, layering the separators with various functional coatings to enhance the overall electrochemistry of the device. Therefore, this research reviews and discusses the application of ALD in SC fabrication and the corresponding improvements achieved in various performance parameters with relation to transition metal compounds.

### 3.1 Nanoparticle Decoration

Transition metal oxides (TMOs) are one of the most optimal candidate materials owing to their high theoretical capacity aided by the redox activity and natural abundance.^[^ ^49^, ^50^, ^206^, ^207^, ^208^, ^209^, ^210^, ^211^, ^212^ ^]^ To this end, coating the nanosized particles and/or films of active materials on high conductivity hosts, e.g., various types of carbon materials, is the most widely adopted strategy. In this architecture, the ultrasmall active materials permit electron exchange with the conducting host.^[^ ^77^, ^213^, ^214^, ^215^ ^]^Furthermore, the advantages of high capacitance of these materials can be leveraged only with nanosized particles or ultrathin films at sizes typically below 10 nm. In this perspective, the inherent advantages of ALD have been extensively explored to obtain a wide variety of TMO nanostructures. For instance, Sun et al.^[^ ^216^ ^]^ prepared TiO_2_–graphene (TiO_2_–G) nanocomposites by depositing TiO_2_ nanoparticles on porous graphene (PGr) sheets via ALD processing of titanium tetrachloride (TiCl_4_) and H_2_O, as a potential SC material. A typical TiO_2_ ALD process is performed with a GPC of ≈0.6 Å at 180 °C, described as follows

(3) $$\begin{array}{ccc} & & n(-\mathrm{OH})\left(s\right)+\mathrm{TiCl}4\left(g\right)\to {\left(-O-\right)}_{n}{\mathrm{TiCl}}_{4-n}\left(s\right)+\mathrm{nHCl}\left(g\right)\\ & & \;\to {\left(-O-\right)}_{n}\cdot {\mathrm{TiCl}}_{4-n}\left(s\right)+(4-n)H_{2}O\left(g\right)\\ & & \;\to {\left(-O-\right)}_{n}\mathrm{Ti}{\left(\mathrm{OH}\right)}_{4-n}\left(s\right)+(4-n)\mathrm{HCl}\left(g\right) \end{array}$$

The gas‐phase deposition reactions in ALD growth facilitate precursor diffusion inside the inward nanopores of the Gr matrix. This type of diffusion is not achieved in conventional deposition techniques. The diameter of the TiO_2_ particles obtained after 50 and 100 ALD cycles was determined as ≈6 nm and ≈10 nm, respectively. The authors demonstrated an increase in the specific capacitance values with the performed ALD cycles to deposit the TiO_2_ nanoparticles. Additionally, the 3D open channels in the composite provided efficient utilization of TiO_2_. Benefitting from these synergistic effects of high conductivity and void spaces for ion movement, the TiO_2_–G composites offered 75 and 84 F g^–1^ at 10 mV s^–1^ as specific capacitances for composites obtained after 50 (TiO_2_ particle diameter: ≈6 nm) and 100 (TiO_2_ particle diameter: ≈10 nm) ALD cycles, respectively.^[^ ^241^ ^]^ In particular, the ALD‐grown TiO_2_–G composite demonstrates satisfactory cycle behavior for over 1000 charge/discharge cycles performed at 2 A g^–1^ and retained 87.5% of the initial capacitance. Therefore, employing ALD to produce composites with highly porous Gr structure can yield hybrid electrode structures that offer much higher energy/power‐density levels and can expand next‐generation power‐storage devices.

The intrinsic defects in the structure of GO/rGO reduce its conductivity and deteriorate the electrochemical performance, thereby limiting their application in the pristine form.^[^ ^217^, ^218^ ^]^ ALD offers unique convenience to accurately functionalize the defects of GO/rGO. Yang et al.^[^ ^219^ ^]^ demonstrated the ability of ALD to achieve highly efficient functionalization. They employed ALD for the precise binding of low quantities of RuO_2_ on rGO. The RuO_2_ loading was controlled via modifying the deposition cycle and coating the inherent defect spots of rGO with RuO_2_ defects. Accordingly, RuO_2_–ALD was performed using Ru(EtCp)_2_ [bis‐(ethylcyclopentadienyl)ruthenium] and oxygen to functionalize rGO with RuO_2_. Generally, ALD can deposit a RuO_2_ thin‐film and RuO_2_ growth has been observed on certain regions. This behavior is attributable to the chemical inertness of Gr and can be ascribed to the chemical activity between the precursor and Gr, because the ALD precursor requires active sites to be chemisorbed during the first half‐reaction. With the binding of Ru to these defects, the substrate undergoes chemical modification in which the C–O sites are initially substituted with C–O–Ru bonds, and subsequently, the growth of RuO_2_ occurs. The nature of these bonds strongly influences the synergy between the materials. Owing to the best synergistic effects, the resultant composite offers a specific capacitance of 1132 F g^–1^ at a scan rate of 50 mV s^–1^, which is proximate to the theoretical limit. A conventional two electrode configuration reveals ≈40% enhancement in the specific capacitance of rGO electrode for an optimal loading of Ru of ≈9.3 wt%. Moreover, the resulting composite electrodes demonstrate significantly improved cycling stability (capacitance retention of ≈92% after 4000 electrochemical cycles), which can be attributed to the close chemical contact of the RuO_2_ particles with the rGO sheet.

As a vital TMO, nickel oxide (NiO) exhibits extremely high theoretical specific capacitance value (2573 F g^–1^), low cost, and environment affability.^[^ ^220^, ^221^ ^]^ Nonetheless, the practical applications of NiO are severely restricted because of its low electronic conductivity and dismal long‐term cycling stability. To improve these aspects, a novel ALD process has been demonstrated in ref. [222] to fabricate NiO/nanoporous graphene (NG) composite as an efficient material for SC electrodes.^[^ ^245^ ^]^ The NiO–ALD deposition has been achieved inside a closed‐type ALD device using nickelocene (NiCp_2_) and O_3_ as precursor materials at 300 °C. The TEM micrographs prominently revealed that the NiO–ALD deposition process preserves the nanoporous nature of the NG substrate. The CV curves of all the prepared NiO/NG nanocomposites revealed a single redox couple, where the anodic peak represents the oxidation of NiO to NiOOH, whereas the cathodic peak indicates the reverse process, expressed as follows^[^ ^222^ ^]^

(4) $$\mathrm{NiO}+OH^{-}+e^{-}↔\mathrm{NiOOH}+e^{-}$$

The NiO/NG hybrid exhibited a high specific capacitance of ≈1005.8 F g^–1^ at 1 A g^–1^ for the total electrode mass (≈1897.1 F g^–1^ for NiO), and stable cycling for 1500 cycles. The performance of the obtained NiO/NG nanocomposites is remarkably influenced by the number of ALD deposition cycles employed to deposit NiO. The cycling behavior of the 500‐NiO/NG composite electrode at 2 A g^–1^ over repeated CD cycle, which indicates the excellent cycling stability of the 500‐NiO/NG electrode, with 94% of the initial capacitance maintained after 1500 cycles.

**Figure 6:**
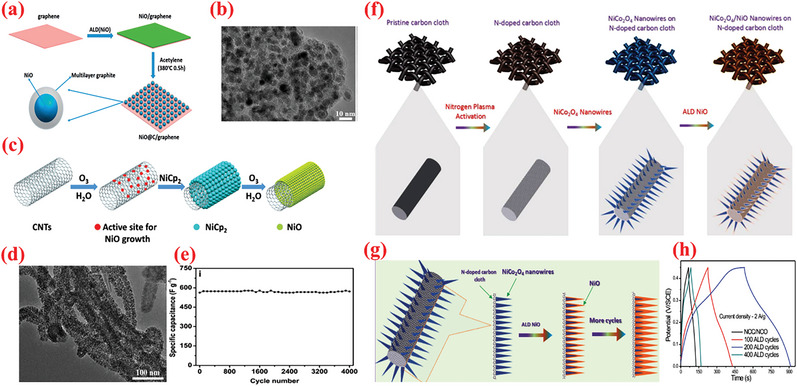
a) Schematic of fabrication of NiO@C/graphene material. b) TEM images of 400‐NiO@C/graphene sample. Reproduced with permission.^[^ ^220^ ^]^ Copyright 2016, Royal Society of Chemistry. c) Graphical illustration of the assumed ALD process mechanism of NiO NPs on CNT. d) TEM images of 200‐NiO/CNT sample. e) Cycling performance of 200‐NiO/CNT sample tested at 10 A g^−1^. Reproduced with permission.^[^ ^223^ ^]^ Copyright 2016, Royal Society of Chemistry. f) Schematic illustration to prepare NCO/NiO C‐S NWs over NCC substrate. g) Schematic displaying the effect of NiO ALD deposition cycles on NCO NWs. h) Comparison of CD profiles for NCC/NCO/NiO C‐S NWs samples with varying ALD‐NiO cycles/layers on NCO NWs. Reproduced with permission.^[^ ^224^ ^]^ Copyright 2019, Wiley VCH.

To improve the high‐rate performance of the NiO‐based electrodes, Yu et al.^[^ ^220^ ^]^ employed the ALD process to decorate graphene with carbon‐coated NiO nanoparticles (NiO@C/Gr) instead of the pristine NiO nanoparticles (**Figure** **6**a–b). They used the same precursor as in ref. [222], followed by annealing at 380 °C in presence of acetylene. The authors further investigated the structure and morphology of the pristine graphene, 400‐NiO/graphene composite, and 400‐NiO@C/graphene composite using TEM analysis. A HR‐TEM image of a distinct particle displayed that the carbon layer was approximately 4 nm thick and covered the crystalline NiO, as depicted in Figure 6b. In reality, the growth temperature poses a significant impact on the sample structures. As discovered, the sample with 400 cycles exhibited a higher specific capacitance than the others, iterating the strong synergy between the NiO concentration and carbon/graphene in their sample. Both carbon layer and graphene in NiO@C/Gr composite‐based SC electrode acted in synergy to offer a high capacitance of 408 F g^–1^ at 1 A g^–1^, excellent rate performance (maintaining 68% even at 50 A g^–1^), and outstanding cyclability (for 2000 cycles at 10 A g^–1^.^[^ ^220^ ^]^

In addition to Gr, CNT is a widely employed carbon allotrope that imparts high conductivity, high surface area, adequate mechanical strength, and chemical stability to SCs.^[^ ^104^, ^225^ ^]^ In this hybrid nanostructured, the size and distribution of the metal oxide MO NPs on the CNTs surface strongly influence the performance of the produced electrode. ALD appears as the most promising technique for controlled synthesis of such NP/CNT hybrids with considerable precision.^[^ ^69^, ^200^ ^]^ Herein, the authors applied O_3_ as the coreactant during the ALD deposition of NiO on CNT surfaces.^[^ ^223^ ^]^ In comparison to water—–the most commonly employed oxygen source during an ALD processing, O_3_ is a stronger oxidizing agent. Moreover, in contrast to H_2_O, the O_3_‐based NiO–ALD process yields growth of NPs only on the impurity and/or defect sites of the CNTs. For ALD–NiO thin‐film deposition, O_3_/H_2_O precursors have been utilized as the only oxygen source. However, nickelocene (NiCp_2_) can be used as the Ni source to produce NiO films at a low growth rate of 0.2 Å per cycle.^[^ ^223^ ^]^ To enhance the ALD growth rate, conductivity, rate, and cycling performance of NiO‐based electrode materials, Wang et al.^[^ ^223^ ^]^ simultaneously employed two oxidizing precursors– – O_3_ and H_2_O– — ‐during a single NiO ALD cycle to prepare NiO/CNT hybrid structures by depositing a uniform NiO coating on the CNTs through simultaneous application of two oxidizing agents: O_3_ and H_2_O (Figure 6c–e). Upon employing both the oxygen precursors simultaneously, the authors achieved a highly conformal NiO coating over the surface of CNTs along with a high growth rate (0.3 Å per cycle). Owing to uniform structure, high electric conductivity, and enhanced NiO–CNT contact, the NiO/CNT hybrid with 200 ALD cycles exhibited a high specific capacitance (622 F g^–1^ at 2 A g^–1^; 2013 F g^–1^ for NiO), superior rate performance (retaining 74% at 50 A g^–1^), and appropriate cyclic behavior for over 4000 discharge–charge cycles. In another study, Ready et al.^[^ ^226^ ^]^ employed four methods to enhance the specific capacitance of CNT‐based devices: room‐temperature ionic liquids (RTIL), functionalizing CNTs by ALD decoration of pseudocapactive materials, plasma‐enhanced CVD driven graphenation of CNTs (gCNT), and integrating the SCs with Si wafer to avoid the use of separator. The functionalization by ALD of TiO_2_ provided pseudocapacitive mechanism, whereas graphenation increased the electrode surface area. The specific energy densities obtained for bare CNTs, gCNTs, and CNTs with TiO_2_ were 2.6, 26.0, and 39.4 W h kg^–1^, respectively. Furthermore, the combination of both graphenation and treatment with TiO_2_ considerably increased the improved energy density (63.4 W h kg^–1^).^[^ ^226^ ^]^

Compared with metal‐based current collectors, carbonaceous current collector such as graphite paper and carbon cloth (CC) offer several benefits such as low density, high porosity as well as 3D structure, thereby rendering them promising for producing high energy and lightweight supercapacitor devices. ^[^ ^227^, ^228^, ^229^, ^230^, ^231^, ^232^ ^]^ Upon utilizing the potential of ALD, Chodankar et al.^[^ ^224^ ^]^ proposed a unique interface engineering strategy employing a suitable combination of ALD and nitrogen plasma to stabilize NiCo_2_O_4_ nanowire/carbon cloth electrodes. First, CC was exposed to nitrogen plasma (NCC) to increase the porosity and introduce nitrogen doping in the carbon microfibers. Thereafter, NCO NW arrays were hydrothermally grown over NCC, followed by the deposition of ultrathin NiO shell using ALD as shown in Figure 6f–h. In particular, the authors used Ni(Cp)_2_ source with O_3_ (4%) reactant; the precursor and line temperature were maintained at 70 and 90 °C, respectively, to ensure the delivery of the source. Based on this recipe, a GPC of 0.03 nm per cycle was determined for the NiO film. Upon depositing the optimized ALD–NiO films on the NCO nanowires for varying numbers of ALD cycles (100–400 ALD cycles), the impact of altering the number of layers of NiO on the SC characteristics was investigated. The optimized NCC/NCO/NiO electrodes containing an over‐layer of NiO with ≈4.4 nm thickness displayed a large capacitance value of 2439 F g^–1^, superior rate (85% in 2–10 A g^–1^ range), and cyclic stabilities. Accordingly, the authors further fabricated foldable solid‐state SCs with an optimized NCC/NCO/NiO electrode. These SCs exhibited remarkable energy density (72.32 Wh kg^–1^) with superior cyclic performance of maintaining 97% capacitance for 10 000 cycles even under varying deformations (i.e., plane and folded states).^[^ ^224^ ^]^ These results indicate the promising potential of ALD‐assisted synthesis of high‐performance nanohybrid electrode materials. The resultant core–shell electrode displayed a remarkably high capacitance value (2439 F g^–1^) along with superior cyclic performance even after 20 000 electrochemical cycles at 4 A g^–1^.

Among various metal oxides, hematite (*α*‐Fe_2_O_3_) is garnering increasing attention owing to its high theoretical capacitance and abundance.^[^ ^233^, ^234^ ^]^ Nonetheless, the inferior electrical conductivity of pristine iron oxides delivers unsatisfactory performance vis‐a‐vis capacitance values and cycling/rate capability. Therefore, increasing the electrical conductivity of iron oxides has evolved as a vital concern. As such, Fe_2_O_3_@MWCNTs nanocomposites prepared by the ALD growth of Fe_2_O_3_ nanocrystallites over the MWCNT surface followed by calcination process demonstrated a specific capacitance of 787 F g^–1^ (1 A g^–1^) with an improved rate (72% capacitance retained or 568 F g^–1^ at 30 A g^–1^) and adequate cyclic property (retaining 91.6% capacitance after 5000 cycles).^[^ ^235^ ^]^ Fe_2_O_3_ film was grown on MWNTs surface via ALD processing of ferrocene (FeCp_2_) and O_3_ at 250 °C. The size of the Fe_2_O_3_ nanoparticles varied between 5 and 10 nm. The authors examined the effects of the Fe_2_O_3_ layer thickness on the electrochemistry of the developed composites by altering the ALD cycle number.^[^ ^235^ ^]^ Their superior performance can be ascribed to the synergistic effects between pseudocapacitive Fe_2_O_3_ NPs (offering large theoretical capacity) and MWNTs (considerably increased the conductivity and surface area). Additionally, bimetallic oxides containing two distinct types of TMOs are suited as pseudocapacitance materials because of their enhanced capacitance resulting from the synergistic effects, increased charge transfer and redox activities. In this perspective, Zhang et al.^[^ ^236^ ^]^ employed ALD to fabricate Co–Ni bimetallic oxides‐coated Ti_3_C_2_T_x_ (CoO_x_NiO/Ti_3_C_2_T_x_) composite electrodes for pseudocapacitors. Bis(cyclopentadienyl) containing ligand for cobalt (CoCp_2_) and nickel (NiCp_2_) precursors have been applied with ozone reactants. The hybrid electrode revealed a uniform coating of metal oxide particles on MXene sheets, which increased the active sites for pseudocapacitive nanoparticles. The storage mechanism of NiO, CoO, and Co_3_O_4_ via hydroxy exchange can be well‐understood by following reactions^[^ ^236^ ^]^

(5) $$\mathrm{NiO}+OH^{-}↔\mathrm{NiOOH}+e^{-}$$

(6) $$\mathrm{CoO}+OH^{-}↔\mathrm{CoCOOH}+e^{-}$$

(7) $${\mathrm{Co}}_{3}O_{4}+OH^{-}+H_{2}O↔3\mathrm{CoOOH}+e^{-}$$

(8) $$\mathrm{CoOOH}+OH^{-}↔{\mathrm{CoO}}_{2}+H_{2}O+e^{-}$$

Consequently, the film deposited with 90 ALD cycles exhibited the most optimal electrochemistry with a remarkably high specific capacitance of 1960 F g^–1^ (at 1 A g^–1^, 20.3 times greater than bare Ti_3_C_2_T_x_), high rate (87.3% retention within 1–8 A g^–1^) and cyclic stabilities (retained 90.2% after 8000 cycles).

**Figure 7:**
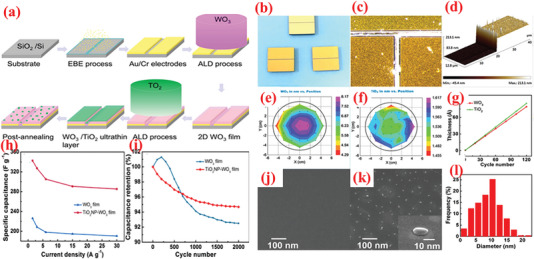
a) The design exemplifies the manufacturing of the TiO_2_NP‐WO3 film used as the electrode for SCs. b) Optical photograph of samples: blank (top), 2D WO_3_ (left), and 2D TiO_2_NP–WO_3_ (right). c) HR Optical photograph of samples: blank (top), 2D WO_3_ (left), and 2D TiO_2_NP‐WO_3_ (right). d) AFM photograph of plane view side between Au/Cr and SiO_2_/Si substrate from blank electrode. Wafer‐scaled ellipsometry measurements for e) 2D WO_3_ and f) 2D TiO_2_. g) Growth rates of WO_3_ and TiO_2_. h) Rate capabilities. i) Cyclic stability test for 2,000 cycles at 6 A g^−1^ over. SEM images of j) 2D WO_3_ film and k) 2D TiO_2_NP‐WO_3_ film after post‐annealing. l) Size distribution histogram of TiO_2_ NPs. Reproduced with permission.^[^ ^237^ ^]^ Copyright 2017, Elsevier

In addition to carbonaceous supports, noncarbon host matrices have been explored to offer enhanced electrochemical performances.^[^ ^238^, ^239^ ^]^ Although WO_3_ with a few layers is a common TMO that has recently been employed in SCs, its reported capacitance values are typically less than that of alternative TMOs. Consequently, researchers are interested to understand the coupling mechanism of thin‐layered WO_3_ with additional materials to improve its performance. For instance, Zhuiykov et al.^[^ ^237^ ^]^ obtained TiO_2_ NPs‐coated 2D WO_3_ nanosheets via a simple two‐step ALD technique and subsequent heat treatment at 380 °C (**Figure** **7**a). Wafer‐scaled WO_3_ films were initially grown on the substrate as part of the ALD process with bis(tertbutylimino)bis(dimethylamino)tungsten(VI) as source of W and H_2_O as source of O.^[^ ^237^ ^]^ In the second step of the ALD process, the 2D WO_3_ film was covered with an ultrathin layer of TiO_2_, wherein the TDMAT and H_2_O vapor was used as Ti and O sources. The heat treatment converted the TiO_2_ layer into NPs. As observed from the optical image of the substrate color in Figure 7b–d, the growth of both materials differed. The thickness of the wafer‐scaled samples measured by ellipsometry are displayed in Figure 7e–g. The TiO_2_ NPs‐coated 2D WO_3_ films demonstrated high improvement (≈1.5 times) in terms of specific capacitance, cyclic stability, rapid charge diffusion rates, and a transition of charge storage mechanism (Figure 7h–i). The SEM analysis displayed that the as‐grown layered WO_3_ films maintained smooth surface after heat treatment, whereas the surface of WO_3_–TiO_2_ film contained tiny NPs after the annealing process, as indicated in Figure 7j–l. This highlights the advantages of ALD over traditional methods for large‐scale deposition of atomically thin layers.

Transition metal carbides (TMCs) are considered a unique class of electrode materials for various applications because of their admirable electric conductivity, toughness, high stability against corrosion, and oxidation among other beneficial properties.^[^ ^47^, ^49^ ^]^ In this regard, Xiong et al.^[^ ^240^ ^]^ synthesized core–shell nickel carbide–carbon nanotube (Ni_3_C/CNT) nanocomposites by conformally depositing Ni_3_C nanoscrystallites (≈12 nm) on porous CNT network via a novel ALD with the precursor bis(1,4‐di‐*tert*‐butyl‐1,3‐diazabutadienyl)nickel(II) and H_2_ plasma. The strong volatility of the nickel diazadienyl precursor facilitated the deposition at a comparatively low temperature of 95 °C. The results revealed that the development of the Ni_3_C films exhibited the ideal characteristic of saturation and self‐limiting growth. The authors prepared a CNT network via a dip‐and‐dry process onto a CC, which was uniformly and conformally coated with a homogenous Ni_3_C layer. When applied as a positive asymmetric electrode (Ni_3_C/CNT//AC), the composite exhibited high specific capacitances of 1850 and 980 F g^–1^ at 2 and 20 mA cm^–2^, and retaining 98.5% capacitance after 5000 cycles, respectively. Using PVA/KOH as a solid electrolyte, the authors assembled flexible asymmetric SCs that exhibited a high energy density of 57 Wh kg^–1^ at a power density of 1.26 kW kg^–1^ and a power density of 12.8 kW kg^–1^ at 35 Wh kg^–1^. Moreover, prolonged cycling stability test performed under various conditions deformations (i.e., normal, bent, and twisted) revealed highly favorable long‐term cyclability while maintaining 98.6% capacity after 50 000 cycles at a high current rate of 16 A g^–1^. The practical viability of the asymmetric supercapacitor has been demonstrated by connecting two cells in series to illuminate a commercial.^[^ ^240^ ^]^

### 3.2 Active/Direct Thin‐Film Deposition

Thin‐films have garnered considerable attention for electrochemical energy storage devices owing to their significantly shorter lengths of charge migration, which improve diffusion kinetics. By leveraging its ability to grow thin‐films with precisely controlled thickness, ALD has been extensively applied for thin‐film deposition, especially to obtain thin‐films on a variety of substrates or surfaces. For instance, Sun et al.^[^ ^216^ ^]^ deposited conformal thin films of amorphous TiO_2_ on graphene surface and multiwalled CNT by ALD using an ultrathin Al_2_O_3_ linkage layer. The nucleation and growth of ALD TiO_2_ occurs only at the defect sites on the surface of G, which results in the distributed deposition of TiO_2_ NPs. To overcome this challenge, generally a thin film of Al_2_O_3_ is predeposited to provide adhesiveness. To develop Al_2_O_3_ adhesion film, the graphene substrate was sequentially exposed to NO_2_ and TMA. The N in NO_2_ acts as a Lewis base and binds with the graphene surface under electron lone‐pair interactions, which creates an abundance of oxygen atoms to react with TMA. Following the treatment with NO_2_, the ALD processing of TMA and H_2_O precursors yielded Al_2_O_3_ following the stated mechanism^[^ ^216^ ^]^

(9) $$\left(A\right)\mathrm{AlOH}*+\mathrm{Al}{\left({\mathrm{CH}}_{3}\right)}_{3}\to \mathrm{AlO}-\mathrm{Al}{\left({\mathrm{CH}}_{3}\right)}_{2}*+CH_{4}$$

(10) $$\left(B\right){\mathrm{AlCH}}_{3}*+H_{2}O\to \mathrm{AlnOH}*+CH_{4}$$

where the asterisk denotes the surface species. An ALD deposition cycle of Al_2_O_3_ comprises the completion of both A and B reactions.

Subsequently, TiO_2_ was deposited over the Al_2_O_3_ adhesion layer via ALD processing of titanium tetrachloride (TiCl_4_) and H_2_O as coreactants

(11) $$\left(A\right)\mathrm{TiOH}*+\mathrm{TiC}l_{4}\to {\mathrm{TiOnTiCl}}_{3}*+\mathrm{HCl}$$

(12) $$\left(B\right)\mathrm{TiCl}*+H_{2}O\to \mathrm{TinOH}*+\mathrm{HCl}$$

One TiO_2_ ALD cycle constitutes both A and B reactions. The ALD‐prepared composites with 50 cycles exhibited high capacitances values of 97.5 and 135 F g^–1^ at 1 A g^–1^.

The improved performance of the CNTs compared to graphene inspired Ready et al.^[^ ^226^ ^]^ to further harness the unique properties of the CNTs to fabricate high‐performance electrode materials. They coated pseudocapacitive TiO_2_ via ALD on vertically aligned multiwalled CNTs (MWCNT) to yield core–sheath structured TiO_2_/MWCNT hybrids and analyzed the influence of the TiO_2_ coating thickness on device performance. The introduction of TiO_2_ with controllable thickness on MWCNT considerably boosted the specific capacitance (>300%) of the hybrid compared with the bare MWCNT electrodes.^[^ ^226^ ^]^ More recently, Yu et al. presented detailed investigations on the effect of ALD of Fe_2_O_3_ films on CNT surfaces using ferrocene (Fe(Cp)_2_), and O_3_ at 200–250 °C.^[^ ^233^ ^]^ The number of ALD growth cycles was varied to control the thickness of the deposited layers (6–30 nm). The authors examined the effect of using various plasma treatments (i.e., pristine, N_2_, and H_2_O) for surface functionalization of nanotubes on the Fe_2_O_3_ coating. The use of ozone as oxidizing species during ALD growth enabled the deposition of Fe_2_O_3_, irrespective of the type of surface treatment. Thus, the ALD‐prepared Fe_2_O_3_‐deposited CNT sponges exhibited a capacitance value of 1000 F g^–1^ at 1 A g^–1^.^[^ ^233^ ^]^ Although the coating Fe_2_O_3_ increased the areal capacitance as expected, a thick coating (30 nm) caused the blockage of active sites with a corresponding deterioration in electrochemical performance.

Vanadium oxides are advantageous for SCs because of their abundant reserves, low cost ($12 kg^−1^), and various oxidation states.^[^ ^113^ ^]^ Specifically, the specific capacitance of vanadium oxide strongly depends on the preparation technique as well as morphology. As V_2_O_5_ is extensively applied in energy storage applications.^[^ ^241^ ^]^ The ALD deposition of V_2_O_5_ over high conductivity and large surface area scaffolds, e.g., CNTs, is widely explored as a potential strategy to enhance its electrochemistry. For instance, Boukhalfa et al.^[^ ^242^ ^]^ fabricated binder‐free flexible composite electrodes comprising ALD‐deposited nanostructured V_2_O_5_ coatings on the surface of MWCNT (V_2_O_5_‐MWCNT) for SC. The authors compared the electrochemical properties of V_2_O_5_‐MWCNT composites prepared after 100, 300, and 500 ALD cycles. The results indicated that the capacitance declined as the number of ALD cycles increased, wherein the composites prepared at 100 ALD cycles exhibited the maximum specific capacitance of 600 F g^–1^ at 1 A g^–1^ and retained 360 F g^–1^ at 20 A g^–1^.^[^ ^242^ ^]^ A vital concern with thicker coatings is the reduction in porosity and consequently surface area. In this perspective, Parsons et al.^[^ ^243^ ^]^ investigated the effects of surface chemistry focusing on the pore structure (meso‐ and microporosity) of carbon electrodes on the ALD nucleation and layer uniformity of V_2_O_5_ in terms of the resultant electrochemical performance. The authors employed two distinct activated carbon (AC) powders as electrodes—labeled as Darco G60 (mesoporous AC) and DLC Supra 50 (microporous C with pore diameter < 11 Å). The ALD procedure utilized vanadium triisopropoxide (VTIP) as a source of V and water vapor via the subsequent half chemical reactions^[^ ^243^ ^]^

(13) $$\begin{array}{ccc} & & {\left(V-\mathrm{OH}\right)}_{\mathrm{surface}}+\mathrm{VO}{\left({\mathrm{OC}}_{3}H_{7}\right)}_{3}\to {\left(O-V-\left({\mathrm{OC}}_{3}H_{7}\right)\right)}_{\mathrm{surface}}\\ & & \;+\mathrm{HO}C_{3}H_{7} \end{array}$$

(14) $${\left(V-\left({\mathrm{OC}}_{3}H_{7}\right)\right)}_{\mathrm{surface}}+H_{2}O\to {\left(\mathrm{VnOH}\right)}_{\mathrm{surface}}+\mathrm{HO}C_{3}H_{7}$$

**Figure 8:**
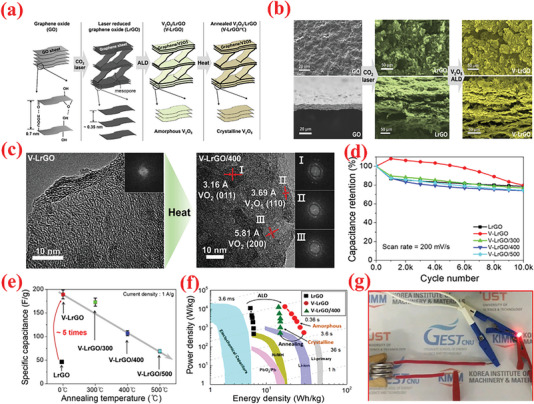
a) Graphical diagram for preparation of multilayered graphene (i.e., LrGO) and amorphous or crystalline V_2_O_5_‐coated LrGO. b) SEM and EDX mapping images of GO, LrGO, and V‐LrGO (V‐LrGO/300cy). c) HR‐TEM images and corresponding FFT images of the V_2_O_5_‐coated graphene before (V‐LrGO) and after annealing at 400 °C (V‐LrGO/400). d) Cycling performance test. e) Specific capacitance variations with different annealed samples. f) Comparison of Ragone plots of prepared symmetric cells with graphene/V_2_O_5_ electrodes with certain literature references. g) Digital image displaying the red mini‐LED lightening. Reproduced with permission.^[^ ^244^ ^]^ Copyright 2020, Elsevier.

The ALD of V_2_O_5_ onto G60 increased the capacitance by ≈46% after 75 cycles of ALD reaction, with the highest capacitance of 540 F g^–1^ obtained with 25 cycles of ALD, small resistance, excellent columbic efficiency, and superior cyclic stability (retaining 89% over 10 000 cycles at 5 A g^–1^). The superior capacitive properties of ALD on G60 carbons reflect the superior ALD coating on the mesoporous carbons.^[^ ^243^ ^]^ The results revealed ALD as a potential candidate technique for constructing high‐performance electrode materials based on ACs for SC applications. Lee et al.^[^ ^244^ ^]^ investigated the consequence of the annealing behavior on the SC product of ALD‐V_2_O_5_‐coated multilayered graphene, as shown in **Figure** **8**a–g. After coating amorphous V_2_O_5_ on multilayered graphene composites (graphene/V_2_O_5_) using low‐T ALD, a series of composite electrodes with distinct crystalline phases was obtained via high‐temperature annealing such as 300, 400, and 500 °C. The results suggested that amorphous V_2_O_5_‐coated graphene composites performed much better as a SC electrode (with respect to higher capacitance value, energy density, and long‐term cyclic performance) compared to the crystalline materials (Figure 8d–g).^[^ ^244^ ^]^

Owing to low electrical conductivity, the application of only MnO_x_ for SC electrode material is highly restricted.^[^ ^25^ ^]^ To this end, MnO_x_ is generally deposited on a high conductivity nanostructured substrate, e.g., CNTs. By varying the ALD process parameters such as the precursor type, oxidant species, and deposition temperature, various phases of MnO_x_ can be obtained. Among the available precursors, methylcyclopentadienyl manganese tricarbonyl (MeCpMn(I)(CO)_3_) is the most widely investigated metal precursor for ALD‐MnO_x_ films owing to its adequately low volatility and thermal stability.^[^ ^248^ ^]^ Pinna et al. employed a novel ALD process for depositing Mn_3_O_4_ using MeCpMn(I)(CO)_3_ as an Mn precursor and ozone as an oxidant. The authors observed saturated growth at short dose pulses (i.e., 0.5 s for MeCpMn(I)(CO)_3_ and 0.2 s for ozone), a moderate GPC of 0.045 nm, and the ability to obtain conformal coating of 3D structures with high aspect ratio. The nanocomposite electrode demonstrated high capacitance values (78.62 mF cm^–2^ at 5 mV s^–1^), which exhibited a higher specific capacitance value compared to only VACNTs. Furthermore, the synthesized VACNTs/Mn_3_O_4_ nanocomposite manifested excellent reversibility and cyclic performance.^[^ ^248^ ^]^

The CoO_x_ (Co_3_O_4_ and CoO) has been explored as a distinctive pseudocapacitive electrode as it affords high specific capacitance combined with low cost and less toxicity.^[^ ^236^ ^]^ High porosity core‐branch nanowire arrays have been reported as intriguing materials for advanced electrodes. Zhang et al.^[^ ^245^ ^]^ successfully fabricated Co_3_O_4_/C nanowires by combining the wet chemical and ALD methods. In this composite, the Co_3_O_4_ nanowire core was homogeneously coated by interconnected amorphous carbon nanoflakes, creating Co_3_O_4_/C core‐branch nanowires with diameters of ≈280 nm. When employed as a SC electrode, it delivered a satisfactory level of specific capacitance (705 F g^–1^) with excellent retention (94%) over 5 000 electrochemical cycles.^[^ ^245^ ^]^ In other work, Guan et al.^[^ ^249^ ^]^ synthesized flexible electrodes via ALD by depositing Co_3_O_4_ nanolayers over CNTs/CC substrate (CNTs@Co_3_O_4_/CC). Influenced by the highly uniform and conformal assembly of Co_3_O_4_ NPs inside the hierarchical architecture, the CNTs@Co_3_O_4_/CC demonstrated a large areal capacitance (≈347.2 mF cm^–2^, ≈10 times more than that of CNTs/CC) with ultrastable cycling performance (no capacity fade after 50 000 cycles).^[^ ^249^ ^]^ The highest capacitance (416.7 mF cm^–2^) was obtained for CNT@2400‐Co_3_O_4_/CC. The increased capacitance for larger number of ALD cycles can be associated with the increase in thickness of Co_3_O_4_ from ≈5 nm for 800 ALD cycles to ≈20 nm for 2400 ALD cycles, including the contribution of Co_3_O_4_ particles (< 20 nm) toward the overall device capacitance.^[^ ^249^ ^]^

Owing to their highly flexible nature, 2D nanomembrane (NM)‐based structures are gaining increasing attention as electrodes.^[^ ^73^, ^250^ ^]^ Supported by enormous surface area, controllable thickness, and highly conformal growth, the ALD approach stands out among them for its clear benefits in the fabrication of NMs.^[^ ^246^, ^247^ ^]^ The recurrent surface interaction of two precursors ensures the deposition of high‐quality, homogeneous NMs. Recently, Huang et al.^[^ ^246^ ^]^ employed ALD to fabricate 2D TiO_2_ NMs of varying thicknesses over a 3D porous polymer template using tetrakis dimethlyamide titanium (TDMATi) and water vapor sources for various ALD cycles, e.g., 100, 200, and 400 cycles. Post‐synthesis, a calcination step was performed in presence of O_2_ at 500°C was to remove the porous template and yield the TiO_2_ NMs for application in electrode fabrication.

The ALD process of surface reaction is generalized by following two‐half chemical equations^[^ ^246^ ^]^

(15) $$\begin{array}{ccc} & & \left(A\right)\mathrm{Ti}{\left(N{\left({\mathrm{CH}}_{3}\right)}_{2}\right)}_{4}+\mathrm{Ti}O_{2}-\mathrm{OH}*\\ & & \;\to N{\left({\mathrm{CH}}_{3}\right)}_{2}+\mathrm{Ti}O_{2}-O-\mathrm{Ti}{\left(N{\left({\mathrm{CH}}_{3}\right)}_{2}\right)}_{3}* \end{array}$$

(16) $$\begin{array}{ccc} & & \left(B\right){\mathrm{TiO}}_{2}-O-\mathrm{Ti}{\left(N{\left({\mathrm{CH}}_{3}\right)}_{2}\right)}_{3}*+2H_{2}O\\ & & \;\to {\mathrm{TiO}}_{2}-\mathrm{Ti}O_{2}-\mathrm{OH}*+3\left(\mathrm{NH}{\left({\mathrm{CH}}_{3}\right)}_{2}\right) \end{array}$$

The TiO_2_ NM optimized at ≈15 nm thickness (100 ALD cycles) generated a maximum capacitance value (2332 F g^–1^) with high energy density (81 W h kg^–1^). The high electrochemical result can be attributed to design strategy, high surface area, and the interconnectedness in ultrathin and flexible NMs.^[^ ^246^ ^]^ Finally, a series combination of two SCs was connected to power one LED of ≈1.5 V, indicating the practical viability of the proposed NMs.^[^ ^246^ ^]^ Similarly, Naeem et al.^[^ ^247^ ^]^ prepared ZnO nanomembranes using 100 ALD‐cycles on polyurethane sponge for electrochemical SC applications.^[^ ^247^ ^]^ The existing ALD‐based grown approach offers a straightforward method to explore the effect, which has been inspired by earlier work by the author that demonstrated the significant impact of the evolving thickness on the ZnO NMs across 50 (8.7 nm), 100 (17.4 nm), and 200 ALD‐cycles (34.8 nm). ALD‐ZnO process was used with DEZ and water vapor with the layer‐by‐layer development of ZnO as shown in the following reaction scheme^[^ ^247^ ^]^

(17) $$\mathrm{Zn}{\left(C_{2}H_{5}\right)}_{2}+H_{2}O\to \mathrm{ZnO}+2CH_{6}$$

The deposited samples were subjected to a 3‐h calcination at 700 °C in an O_2_ environment to ensure the removal of ZnO NMs from the sponge template. The CVs of these composites exhibited deviations from the perfect rectangular profile and displayed redox peaks, thereby signifying the pseudocapacitive charge‐storage behavior.^[^ ^247^ ^]^ The ZnO NMs were electrochemically characterized in various electrolytes such as KOH, KCl, and Na_2_SO_4_, with 6 m KOH delivering optimum performance with a high capacitance (846 F g^–1^ at 1 A g^–1^ and 92 F g^–1^ at 5 A g^–1^) and remarkable cyclic stability for 5000 cycles. Additionally, SCs assembled using the prepared ZnO NMs exhibited immense potential in practical applications by flashing a red LED for 180 min.^[^ ^247^ ^]^ Utilizing the properties of ALD for depositing highly conformal and uniform nanomembranes, Zhao et al.^[^ ^251^ ^]^ integrated a metal–oxide framework (ZIF‐8) over a high‐mass carbon foam (CF) surface loaded with efficiently inducting ALD‐produced ZnO nanomembrane. Subsequently, the pyrolysis step yielded a stretchy N‐doped carbon particle‐CF (N‐CP‐CF) with a porous construction and high active area.^[^ ^251^ ^]^ Using a home‐made reactor, ZnO NM was applied to the CF skeletons at 150 °C, and DEZ and H_2_O vapor were utilized as precursors. The composite offered high capacitance values (300 F g^–1^ at 0.5 A g^–1^). Furthermore, after 25% compression, the capacitance slightly decreased to 250 F g^–1^.^[^ ^251^ ^]^ In particular, a symmetric SC exhibited an energy density of 20.8 and 3.6 W h kg^–1^ at power densities of 250 and 10 000 W kg^−1^, respectively. The use of ALD for ZIF‐8 deposition provided a uniform coverage of the CF, which enhanced the SC performance.

In addition to TMO and TMC, TM nitrides (TMNs) have recently emerged as attractive, versatile electrode materials for SC applications owing to a range of favorable properties such as high electronic conductivity, narrow bandgap, adequate chemical and thermal stabilities, high volumetric capacity, and wide abundance.^[^ ^252^, ^253^, ^254^ ^]^

**Figure 9:**
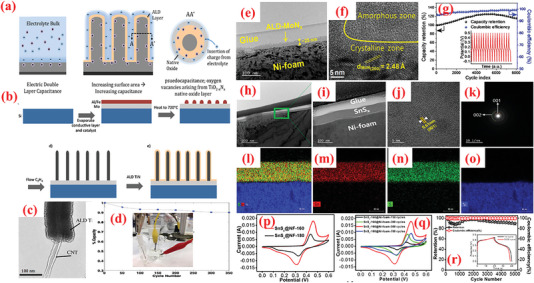
a) Graphical representation of the idea of improving performance by enhancing surface area and pseudocapacitive behavior. b) Schematic illustration of ALD grown TiN conformally on the CNT array. c) TEM image of ALD grown TiN on CNT displaying conformal coverage with 20 nm. d) Cyclic stability test. Reproduced with permission.^[^ ^256^ ^]^ Copyright 2016, Elsevier. e) Cross‐view TEM (XTEM) image of MoN_x_@NF sample, and f) crystalline and amorphous sections recognized through HR‐TEM investigation. g) Cycling performance with corresponding coulombic efficiency (Inset: remaining CD cycles) for MoN_x_@NF composite. Reproduced with permission.^[^ ^255^ ^]^ Copyright 2018, Elsevier. h–o) XTEM images of SnS_x_@NF‐160 sample prepared using with 500 ALD cycles, and corresponding STEM elemental mapping to confirm the uniformity and conformity deposition on NF. p) Comparative CV profiles. q) comparison of the CV curves plotted with different ALD cycles at 5 mV s^−1^. r) Cyclic stability is demonstrated by capacitance retention and corresponding CE for the prepared electrodes. Reproduced with permission.^[^ ^175^ ^]^ Copyright 2019, Springer Nature.

To exploit the pinhole‐free growth ability of ALD, Kao et al.^[^ ^256^ ^]^ reported high conformality and accurate loading of titanium nitride (TiN) coated on high‐aspect‐ratio vertically aligned CNTs (VACNTs) forest via ALD processing of TDMATi and nitrogen plasma for electrochemical SCs (**Figure** **9**a–d). After 400 ALD cycles, a 20‐nm‐thin film of TiN was uniformly deposited over individual nanotubes in the forest. As SCs electrodes, the TiN–CNT hybrid exhibited 2000 times larger capacitance than the planer TiN electrodes. Additionally, the specific capacitance of TiN‐CNT SC (81 mF cm^–2^) improved by over 500% compared to pristine CNT forest SCs (14 mF cm^–2^). The increased capacitance can be attributed to the i) increased surface area caused by the porous CNT forest design, and ii) enhanced redox activity in oxygen‐deficient TiN film.^[^ ^256^ ^]^ Recently, Ansari et al.^[^ ^156^ ^]^ demonstrated a novel low‐temperature (70–200 °C) ALD procedure to synthesize thin‐films of SnN_x_ using tetrakis(dimethylamino)tin and ammonia. The developed ALD process exhibited typical features of an ideal ALD characteristics with self‐limiting growth. The SnN_x_@Ni exhibited more charge‐storage time (≈8 times greater than that for bare NF). Moreover, the CV curves revealed the presence of both EDLC as well as Faradaic mechanisms. This enhanced performance was ascribed to uniform coating of SnN_x_ on NF, ensuring stable connection and structural stability. Additionally, the electrode offered remarkably stable cycling with ≈92% capacitance retention over 3000 cycles and excellent coulombic efficiency (> 97%).^[^ ^156^ ^]^

Kim et al.^[^ ^255^ ^]^ directly deposited MoN_x_ on 3D Ni‐foam (NF) using Mo(CO)_6_ and NH_3_ at 250 °C via ALD, yielding MoN_x_@NF composite that was directly used as SC electrodes. The MoN_x_@NF composite demonstrated highly uniform and conformal deposition of MoN_x_ thin film (≈25 nm) over NF, as evident from the TEM images mentioned in Figure 9e,f. The CV curves revealed considerable improvements in the charge stored in MoN_x_@NF vis‐a‐vis bare NF electrode. In this composite, NF affected the redox‐reactions and endowed a large areal capacitance (geometrical) of 130 mC cm^–2^ at 2 mA cm^–2^ with excellent cyclic (> 100% retention over 8,000 discharge/charge cycles with ≈100% CE) and rate capability (85% retained from 2 to 10 mA cm^–2^), as displayed in Figure 9g. Similarly, the deposition of RuN_x_ films via ALD on MWCNTs forest demonstrated a high capacitance of 728 F g^–1^ with 1 m KOH as electrolyte.^[^ ^255^ ^]^

**Figure 10:**
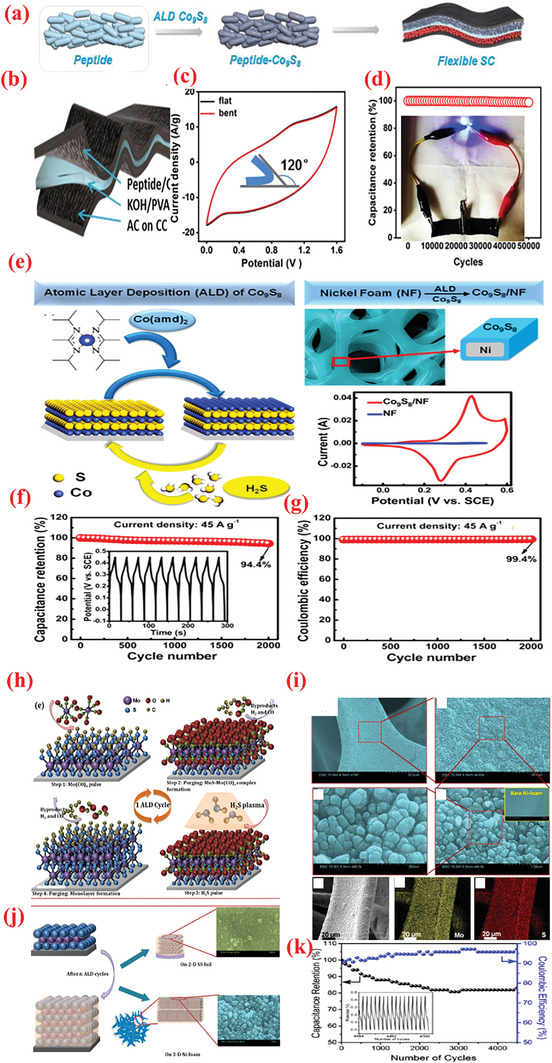
a) Preparation for peptide–Co_9_S_8_ core–shell nanostructures and TENG/SC system. b) Schematic of peptide–Co_9_S_8_//AC flexible solid‐state asymmetric supercapacitor. c) CV curves comparing the flat‐state and bent‐state of the supercapacitor device (bending angle: 120°). Image of LED illuminated using two peptide–Co_9_S_8_//AC supercapacitors in series. d) Long‐term cycling stability test at 16 A g^−1^. Reproduced with permission.^[^ ^258^ ^]^ Copyright 2019, Elsevier. e) Graphic illustration of ALD‐grown Co9S8 on NFs. f) Cycling stability tested at 45 A g^−1^ for 2000 cycles (Inset: CD profiles for first 10 cycles). g) Coulombic efficiency graph during the cycling performance test. Reproduced with permission.^[^ ^259^ ^]^ Copyright 2015, American Chemical Society. h) Schematics of ALD‐MoS_2_ using Mo(CO)_6_ and H_2_S plasma i) SEM micrographs of MoS_2_@3DNF with 400 ALD cycles. EDS mapping of ALD‐MoS_2_ displaying uniform coverage of Mo and S on NF. j) ALD‐MoS_2_ on 2D SS foil and 3D NF substrates. k) charge‐discharge performance for the prepared MoS_2_@3DNF composite using 400 ALD cycles. Reproduced with permission.^[^ ^264^ ^]^ Copyright 2017, American Chemical Society.

Sulfides of TM have been deposited on NF to obtain appropriate electrochemical performance.^[^ ^257^ ^]^ For example, as depicted in **Figure** **10**a, ALD was recently used to grow a conformal layer of Co_9_S_8_ on the peptide nanostructure to create peptide–Co_9_S_8_ core–shell nanobrick hybrids.^[^ ^258^ ^]^ To optimize the thickness of the Co_9_S_8_ layer, the authors varied the number of ALD deposition cycles. The electrode comprising the peptide–Co_9_S_8_ nanobricks composite exhibited a large specific capacitance value of 1300 mF cm^–2^ (1800 F g^–1^) at 0.7 mA cm^–2^ along with a superior cyclic performance with 96% retention after 5000 cyclic processes. Moreover, prolonged cycling tests conducted on the peptide–Co_9_S_8_//AC device at a constant current density of 16 A g^–1^ suggest highly stable cycling performance along with 98% capacitance retention for over 50 000 cycles, thereby suggesting the remarkable cyclic performance of the peptide–Co_9_S_8_//AC device. Finally, the authors integrated the peptide–Co_9_S_8_//AC asymmetric supercapacitor with a TENG to obtain a wearable TENG/SC system (Figure 10b–d).^[^ ^258^ ^]^ The as‐prepared device can supply energy to red LED for 21 min on a 2.7 h charge by TENG, which demonstrates the remarkable self‐charge and energy‐delivery properties of the developed device and displays a promising system for suitable wearable electronic devices. Wang et al.^[^ ^259^ ^]^ reported the superior electrochemical performance of Co_9_S_8_/Ni‐based electrodes prepared by deposition of highly pure Co_9_S_8_ films on NF via ALD using bis(*N*,*N*′‐ diisopropylacetamidinato)cobalt (II) and H_2_S precursors at 120 °C, yielding polycrystalline films with ≈54 nm thickness after 2000 ALD cycles (Figure 10e). The Co_9_S_8_/Ni offered high specific capacitance (1645 F g^–1^ at 3 A g^–1^) with superior cyclability (only ≈5.6% capacitance dissipation over 2000 cycles) and remarkable Coulombic efficiency (> 99.4%) (Figure 10f–g). The research group employed the same ALD process for fabricating a flexible supercapacitor device. Compared to conventional materials, peptide‐based biomaterials are attractive because of their abundant availability, low cost, intrinsic flexibility, structural stability, and versatility.^[^ ^260^, ^261^, ^262^, ^263^ ^]^

Similarly, Nandi et al.^[^ ^264^ ^]^ proposed a binder‐less MoS_2_@3DNi composite prepared by PEALD of MoS_2_ onto 3D Ni foam using molybdenum hexacarbonyl and H_2_S precursors at 200 °C,^[^ ^264^ ^]^ as depicted in Figure 10h–j. The authors thoroughly studied the growth mechanism of MoS_2_ on NF including the properties of the grown films. In addition, they discussed the influence of the MoS_2_ film thickness on the electrochemical characteristics to determine the optimum number of ALD deposition cycles for SC applications. The PEAD growth of MoS_2_ follows the half reactions^[^ ^264^ ^]^

(18) $$\left(A\right)\mathrm{Mo}{\left(\mathrm{CO}\right)}_{x}*+H_{2}S\to \mathrm{Mo}-\mathrm{SH}*+H_{2}\left(g\right)+\mathrm{CO}\left(g\right)$$

(19) $$\begin{array}{ccc} & & \left(B\right)\mathrm{MoSH}*+\mathrm{Mo}{\left(\mathrm{CO}\right)}_{6}\left(g\right)\\ & & \;\to \mathrm{Mo}-S-\mathrm{Mo}{\left(\mathrm{CO}\right)}_{x}*+H_{2}\left(g\right)+\mathrm{CO}\left(g\right) \end{array}$$

where the asterisk (*) presents the surface species.

Compared with ALD‐grown MoS_2_ on flat stainless steel and bare Ni foam‐based electrodes, the MoS_2_@3D‐Ni electrode offered manifold enhancements in capacity. The MoS_2_@3D‐Ni composite prepared with 400 ALD cycles revealed record high areal capacitance (3400 mF cm^−2^ at 3 mA cm^−2^), significantly high‐rate performance (up to 50 mA cm^–2^), long cyclic life (>80% capacity retention), and excellent Coulombic efficiency (≈95%) over 4500 CD cycles (Figure 10k).^[^ ^264^ ^]^ Furthermore, the same research group extended this process to prepare SnS_x_@Ni hybrids by performing ALD of SnN_x_ on 3D Ni foam with TDMASn and H_2_S sources.^[^ ^175^ ^]^ Upon varying the ALD process temperature from 160 to 180 °C, the authors could obtain two distinct types of ALD–SnS_x_ films, i.e., hexagonal SnS_2_ (*h*‐SnS_2_) and orthorhombic SnS. Overall, the films were uniform and conformal over the NF substrate (Figure 9h–o), which displayed the potential of ALD over alternative deposition methods. However, the SnS_x_@NF fabricated at 160 °C offered higher double‐layer capacitance than that grown at 180 °C (Figure 9p–q).^[^ ^175^ ^]^ Moreover, the authors studied the effects of varying the number of ALD cycles (150, 300, 500, and 700 cycles) on the electrochemical performance of both electrodes, among which SnS_x_@NF prepared at 160 °C with 500 ALD cycles displayed optimized performance (Figure 9r). The optimized electrode with a 60‐nm‐thick SnS_x_ coating exhibited an aerial capacitance of 805.5 mF cm^–2^ at 0.5 mA cm^–2^ current rate, appropriate cycling behavior (> 90% retention for 5000 cycles), and high Coulombic efficiency (≈99%).^[^ ^175^ ^]^ Recently, Pawar et al.^[^ ^265^ ^]^ demonstrated the electrochemical performance of a composite SC electrode prepared by depositing MoS_2_ thin‐film over high‐surface‐area NiCo_2_O_4_ nanosheets, grown over Ni‐foam, via ALD. The authors compared the performances of the MoS_2_‐coated NiCo_2_O_4_ (NiCo_2_O_4_–MoS_2_) and MoS_2_‐coated Co_3_O_4_ (Co_3_O_4_–MoS_2_) electrodes, implying the advantages of highly conducting sulfide layer and the superiority of ternary NiCo_2_O_4_ over binary Co_3_O_4_ for improved performance of the SCs. The as‐prepared sample with MoS_2_ deposited by an optimum number of 500 ALD cycles (i.e., NiCo_2_O_4_–MoS_2_‐500) confirmed the higher areal capacitance (2445 mF cm^–2^) along with the improved rate performance. Lastly, the authors assembled an asymmetric SC device by combining the optimized electrode with AC and exhibited the practical application of the constructed device by illuminating an LED. These studies highlight the advantages of combining ALD with a large‐surface‐area ternary oxide‐based 3D architecture.^[^ ^265^ ^]^ Thus, the combination of ALD with conventional hydrothermal synthesis offers immense potential for obtaining electrodes with improved performance for futuristic SCs. Generally, Faradaic SCs exhibit inferior cyclic stabilities owing to their limited electric conductivity. To enhance the cyclic stability of Faradaic SCs, Fang et al.^[^ ^266^ ^]^ fabricated novel porous carbon (PC) coating with “gap shell” configuration over carbon fiber cloth (CFC)/NiS_2_ (denoted as CFC/NiS_2_/PC) to obtain electrode materials by combining ALD Al_2_O_3_ and molecular layer deposition (MLD) alucone, followed by carbonization and etching. As binder‐free electrode for SCs, the CFC/NiS_2_/PC composite revealed a high specific capacitance (1034.6 F g^–1^ at 1 A g^–1^) with superior rate performance of 67% (1–20 A g^–1^) properties. Over 2000 cycles, the CFC/NiS_2_/PC electrode offered a 50% improvement in cycling stability than CFC/NiS_2_, which is attributable to the gap shell configuration and the conductive PC coating.^[^ ^266^ ^]^

**Figure 11:**
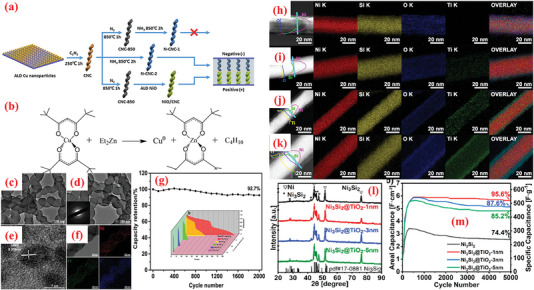
a) Schematic illustration of synthesis of pure carbon nanocoils. b) ligand‐exchange process between Cu(thd)_2_ and Et_2_Zn. TEM images of c) 800‐ and d) 1200‐NiO/CNC. e) HRTEM image f) with elemental mapping of Ni, O, and C for single 1200‐NiO/CNC. g) Cyclic stability test for 2000 cycles at 5 A g^–1^. Reproduced with permission.^[^ ^267^ ^]^ Copyright 2018, Elsevier. HAADF‐STEM images and line profiles with corresponding elemental mappings of a single (h) Ni_3_Si_2_, i) Ni_3_Si_2_@TiO_2_‐1nm, j) Ni_3_Si_2_@TiO_2_‐3nm, k) Ni_3_Si_2_@TiO_2_‐5nm nanowire. l) Cycling performance of Ni_3_Si_2_, Ni_3_Si_2_@TiO_2_‐1nm, Ni_3_Si_2_@TiO_2_‐3nm, and Ni_3_Si_2_@TiO_2_‐5nm fur electrodes at 100 mA cm^–2^. m) XRD patterns of Ni_3_Si_2_ (black), Ni_3_Si_2_@TiO_2_‐1nm (red), Ni_3_Si_2_@TiO_2_‐3nm (blue), and Ni_3_Si_2_@TiO_2_‐5nm (green). Reproduced with permission.^[^ ^268^ ^]^ Copyright 2021, Elsevier.

In addition to using carbon materials as the conducting hosts for high‐capacity materials, various carbon materials have been prepared via ALD with desired properties. For instance, Wang et al.^[^ ^267^ ^]^ adopted a novel high‐yield strategy for fabricating highly pure carbon nanocoils (CNCs) using ALD‐grown Cu nanoparticles as catalysts. The uniformly dispersed Cu nanoparticles were prepared via an ozone deficient ALD process leveraging a facile ligand‐exchange process between Cu(thd)_2_ and Et_2_Zn (**Figure** **11**a,b).^[^ ^267^ ^]^ The synthesis process was conducted following two consecutive saturated‐surface responses: the initial step involved the adsorption of the Cu(thd)_2_ monolayer on the substrate surface via dipole–dipole interactions; the second step involved the transportation of Et_2_Zn and carrier gas inside the reaction chamber, followed by a saturation reaction between Et_2_Zn and Cu(thd)_2_ monolayer to yield elemental Cu.

The scheme in Figure 11a illustrates the detailed procedure of preparing CNCs and its application. The thermal decomposition of acetylene on the catalyst of Cu surface results in the formation of pure CNCs. Additionally, NiO/CNC hybrid structures were prepared via ALD technique using carbonized CNCs as substrates. The morphology of the synthesized Cu films with dense, tiny, and granular crystallites is presented in Figure 11c,d, which demonstrates the ability of the reported method to prepare uniform Cu nanoparticles. The formation of the helical structures can be ascribed to the high‐growth anisotropy of Cu NPs on various crystal planes, primarily because of the large variation in adsorption energy (Figure 11e,f). The heavily doped CNC exhibited optimal electric double‐layer capacitance (143 F g^–1^) compared to the earlier reports of CNC‐based electrodes. Furthermore, the carbonized CNC was decorated with NiO via ALD with NiCp_2_ and O_3_ as precursors to yield NiO/CNC hybrids with higher capacitance (430 C g^–1^) along with appreciable rate and cyclic stabilities (Figure 11g).^[^ ^267^ ^]^ More recently, Zhang et al.^[^ ^268^ ^]^ prepared Ni_3_Si_2_@TiO_2_ furs as a novel composite material for SC electrodes. First, high‐aspect‐ratio Ni_3_Si_2_ nanoarrays with high area density and large exposed area were in situ deposited over Ni foam as furs via a low‐pressure CVD technique. Thereafter, TiO_2_ films with varying thickness were grown over the Ni_3_Si_2_ nanowires via ALD (Figure 11h–l). As observed, the optimized Ni_3_Si_2_@TiO_2_ furs with 1‐nm‐thick TiO_2_ coating exhibited an extremely large areal capacitance (7.82 F cm^–2^ at 5 mA cm^–2^), superior rate performance (5.91 F cm^–2^ at 100 mA cm^–2^), and excellent cycling stability (95.6% retention over 5000 CD cycles at 100 mA cm^–2^) (Figure 11m). Furthermore, the authors assembled an asymmetric SC device using the optimized electrode, which delivered an energy density of 1.528 mW h cm^–3^ at a power density of 25 mW cm^–3^ and 0.93 mW h cm^–3^ at 0.1 W cm^–3^.

**Figure 12:**
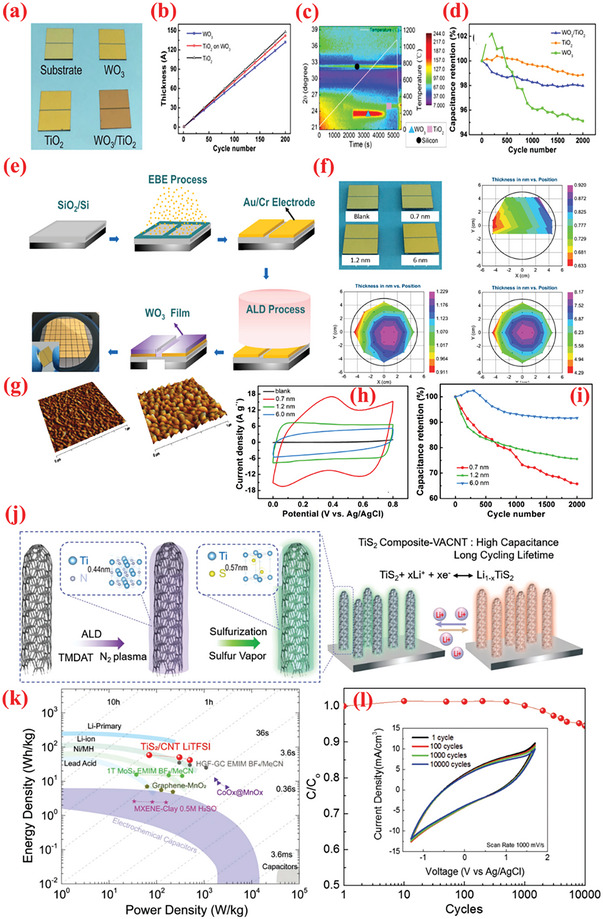
a) Optical picture. b) Growth rate confirmation. c) XRD analysis, d) Long‐term cyclability of three 2D NMs at 6 A g^–1^. Reproduced with permission.^[^ ^271^ ^]^ Copyright 2017, Elsevier. e) Synthesis protocol of 2D WO_3_. f) Digital photographs and wafer‐scaled ellipsometry measurement. g) AFM images of 2D WO_3_ films with thickness of 0.7 nm (left) and 6.0 nm (right). h–i) CV curves and cyclic stability of samples with electrodes of varying thicknesses. Reproduced with permission.^[^ ^272^ ^]^ Copyright 2017, Elsevier. j) TiS_2_–VACNT hybrid nanocomposite fabrication by two‐step process: ALD grown TiN coated on VACNT and transformed to TiS_2_ complex in S atmosphere. Enhanced capacitance values in Li^+^ electrolyte realized via EDLC and TiS_2_ composite–Li intercalation. k) Ragone plot showing the highest energy density among several noncarbon materials. l) Cyclic stability test. Reproduced with permission.^[^ ^273^ ^]^ Copyright 2018, Wiley‐VCH.

Although the effects of the electrode thickness on the electrochemical performance of a supercapacitor are well established, it has not yet been explored at the nanoscale.^[^ ^269^, ^270^ ^]^ To this end, a few studies have investigated these effects at the submicron‐scale on dozens of nanometers. For instance, Hai et al.^[^ ^271^ ^]^ demonstrated the potential of wafer‐scale WO_3_/TiO_2_ heterojunction (HJ) prepared through ALD process with similar recipe for SC electrodes. The grown WO_3_/TiO_2_ HJ exhibits a rough surface and a consistent thickness of ≈12 nm. Owing to the varying thickness of the deposited nanomaterials, the color of WO_3_/TiO_2_ HJ is significantly darker than that of TiO_2_ and WO_3_, and both these materials appear slightly darker in contrast to a blank electrode (**Figure** **12**a–c). The GPCs of 0.66, 0.74, and 0.71 Å per cycle for WO_3_, TiO_2_, and TiO_2_ on WO_3_ surface were obtained through ellipsometry analysis (Figure 12b). The as‐prepared WO_3_/TiO_2_ HJ electrode exhibited high SC performances in terms of high specific capacitance (625.53 F g^–1^ at 1 A g^–1^) and longer cyclic stability for 2000 cycles with 97.98% retention (Figure 12d). The findings of this study may provide fresh avenues for investigating further HJs created via ALD for other potential applications. Therefore, this inquiry might serve as a general example of fabricating 2D heterojunctions on a vast scale for high‐performance SCs. The same group of Zhuiykov et al.^[^ ^272^ ^]^ investigated the effect of the thickness of ALD‐deposited 2D WO_3_ electrodes on the SC performance in the nanoscale regime. To study the nanoscale effect, three atomically thin 2D WO_3_ electrodes were fabricated by varying the film thickness from ≈6.0 to ≈0.7 nm, corresponding to nearly nine layers in comparison to a single layer (monolayer) of WO_3_. The schematic of the synthesis process of 2D WO_3_ SC electrodes is illustrated in Figure 12e. As observed from the figure, the color of 2D WO_3_ electrode varies from pale to bright yellow with an increased film thickness, which was consistent with WO_3_ stoichiometry.^[^ ^272^ ^]^ Nonetheless, as depicted in Figure 12f–g, the influence of these factors strengthened over the film uniformity as the film thickness increased, especially the temperature gradient between the middle of the wafers toward the edges. The origin of pseudocapacitance can be associated with the reaction: WO_3_ + e^–^ + H^+^ → HWO_3_, where H^+^ ions transfer at the monolayer WO_3_ surface results in the redox process (W^6+^ to W^5+^),^[^ ^272^ ^]^ as depicted in Figure 12h. The reduction in the 2D WO_3_ film thickness significantly enhanced the capacitance values (225.4–650.3 F g^–1^); however, its rate performance declined from 83.9% to 65.4%. Additionally, as the film thickness diminished, the capacitance retention decayed from 91.7% to 65.8% along with inferior cycle performance for 2000 cycles (Figure 12i).

As the most lightweight and affordable TM dichalcogenides (TMDCs), TiS_2_ offers the following advantages:^[^ ^274^, ^275^ ^]^ a) high energy density, b) rapid ion transport, c) relatively less volume fluctuations induced by CD cycling, and d) no phase transformation. Nevertheless, TiS_2_ is plagued by low electrical conductivity and mechanical breakdown during deionization and ionization. To overcome these challenges, the conformal deposition of TiS_2_ on 3D structures such as VACNTs can potentially increase its electronic conductivity, high surface area, and mechanical strength. For instance, by uniformly depositing TiS_2_ on VACNTs, Zang et al.^[^ ^273^ ^]^ extended the working voltage to > 3 V, thereby entailing a high specific capacitance value of 195 F g^–1^ with an Li‐rich electrolyte (Figure 12j). Nonetheless, the conventional ALD of TiS_2_ employs TiCl_4_ and H_2_S, which is highly toxic and releases sulfur contaminating the ALD reactors. Furthermore, the nanostructures obtained via ALD/sulfurization process exhibit improved charge transfer and surface reactions, resulting in a high‐power density (1250 W kg^–1^) with 10 000 cyclic stability(Figure 12k).^[^ ^273^ ^]^ The electrode exhibited a small distortion ratio along with a high capacitance retention (≈95%) after 10 000 CD process in CV tests (Figure 12l).

### 3.3 Multimetal and Hybrid Electrodes

**Figure 13:**
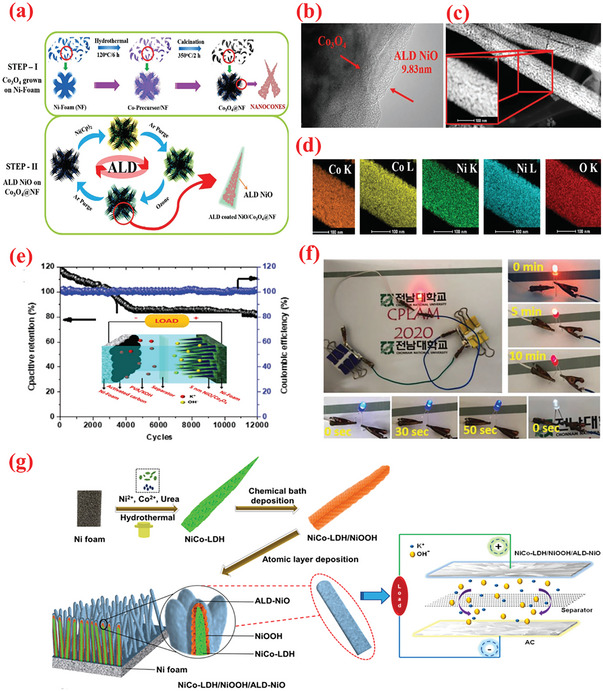
a) Schematic of synthesis procedure of Co_3_O_4_ nanocone arrays (Co_3_O_4_@NF) and ALD NiO on Co_3_O_4_@NF. b–d) STEM micrograph of ALD 5 nm NiO/Co_3_O_4_@NF, corresponding to elemental mapping of elements d) Co, Ni, and O. e) Cyclic stability and coulombic efficiency during 12 000 cycles at 5 A g^–1^ of 5‐nm‐thick NiO/Co_3_O_4_@NF||AC asymmetric SC. f) Digital images of assembled solid‐state asymmetric SC illuminating LEDs (red, light, and white). Reproduced with permission.^[^ ^280^ ^]^ Copyright 2020, Wiley‐VCH. g) Schematic protocol of NiCo‐LDH/ NiOOH/ALD‐NiO C‐S NWs on 3D NF. Charge storage process of AC anode and CNN heteronanostructure cathode in the two‐electrode system. Reproduced with permission.^[^ ^281^ ^]^ Copyright 2021, American Chemical Society.

To increase the energy density of SCs, pseudocapacitive binary and mixed MOs have been extensively explored because of their higher theoretical capacitances, affordability, and redox activity vis‐a‐vis monometal oxides.^[^ ^276^, ^277^, ^278^, ^279^ ^]^ In this regard, Kim et al. recently demonstrated core–shell structured free‐standing NiO/Co_3_O_4_ nanocone arrays on the Ni‐foam substrate to yield a hybrid SC electrode (NiO/Co_3_O_4_@NF) with high capacitance (**Figure** **13**). First, high‐aspect‐ratio Co_3_O_4_ nanocones were deposited on NF via hydrothermal reaction and subsequent ALD growth of NiO shell over Co_3_O_4_ nanocone as the core materials (Figure 13a). In this design, Co_3_O_4_ acts as a host for the atomically thin NiO layer and NiO coating conforms to the shape of the Co_3_O_4_ host, thereby constituting a configuration similar to the core–shell structure. The authors investigated the effects of NiO layer thickness (3, 5, 7 and 10 nm) on SC performance by varying the number of ALD growth cycles (Figure 13b–d). Consequently, the hybrid comprising an optimized 5‐nm‐thick NiO shell exhibited a maximum specific capacitance of 1242 C g^–1^ (2760 F g^–1^) at a current density 2 A g^–1^ as compared to pure Co_3_O_4_@NF (1045.8 C g^–1^ or 2324 F g^–1^). This sample demonstrated extended rate (≈77% or 959.8 C g^–1^ retained at 10 A g^–1^) and cycling (95.5% cyclic stability over 12 000 cycles at 5 A g^–1^) stability compared to Co_3_O_4_@NF electrode (with 46% capacitance retention at the same current) (Figure 13e). Additionally, full‐cell asymmetric SC was assembled using the 5 nm NiO/Co_3_O_4_@NF as cathode, AC as anode, cellulose separator, and PVA‐KOH gel electrolyte, yielding energy densities of 81.45, 68.01, 45.33, and 28.12 W h kg^–1^ at power densities of 4268, 5112, 6771, and 8578 W kg^–1^, with adequately cycling stability of the device. Finally, the practical utility of the assembled asymmetric SCs was tested by illuminating multicolored LEDs (2.0 V) using a series combination of two SCs. Upon charging for 5 min, the LED glow was maintained for >5 min in dark (Figure 13f).

Nevertheless, most hybrid electrodes are susceptible to physicochemical degradation that results in unsatisfactory cycling performances.^[^ ^282^, ^283^, ^284^ ^]^ In this regard, coating the core–shell structure with a thin protective layer is a beneficial strategy that can improve the chemical as well as physical stability of the electrode architecture. For instance, Kavinkumar et al.^[^ ^285^ ^]^ encapsulated core–shell NiCo_2_O_4_/MoO_2_ heteronanostructure within an ALD‐grown NiO nanolayer and investigated the potential SC electrodes via both theoretical and experimental analyses. First, NiCo_2_O_4_ was hydrothermally grown over NF, followed by another hydrothermal step to obtain NiCo_2_O_4_/MoO_2_, which were thereafter encapsulated by the NiO layer via ALD at 175 °C. The exterior NiO layer preserved the structural integrity of the underlying NiCo_2_O_4_/MoO_2_ heteronanostructure by preventing cycling‐induced pulverization as well as offered additional capacity of >450 C g^–1^. The flexibility provided by the NiO layer reduced the capacity loss to < 3% compared to that for noncoated electrodes (10%) after 20 000 cycles. Finally, asymmetric SCs were assembled with NiCo_2_O_4_/MoO_2_@ALD‐NiO as the affirmative and a B‐doped RGO‐coated with ALD‐Fe_2_O_3_ as the negative electrode to form NiCo_2_O_4_/MoO_2_@ALD‐NiO || B‐RGO@ALD‐Fe_2_O_3_, which delivered a high energy density (136 W h kg^–1^ at 1800 W kg^–1^), high power density (101 W h kg^–1^ at 18 000 W kg^–1^), and adequate rate performance.^[^ ^285^ ^]^ The cyclability of the as‐fabricated ASC device after 20 000 cycles at 10 A g^–1^ demonstrated 92.3% capacity retention over 20 000 GCD cycles, thereby revealing adequate stability and long cycling lifespan.^[^ ^285^ ^]^ The same research team synthesized a NiCo‐LDH/NiOOH/ALD‐NiO with a core–double‐shell structure in which a thin film of ALD–NiO acted as a support to NiOOH to stabilize the electrode during tests, thereby improving the performance.^[^ ^281^ ^]^ A 2‐step process of hydrothermal and chemical bath deposition was performed to construct core/shell structures on NF, followed by ALD on the top shell to obtain the core/double‐shell structure, as depicted in Figure 13g. The obtained electrode exhibited an unusual specific capacity (1420.2 C g^–1^ at 4 A g^–1^) and reserved 93% capacity even after 20 000 cycles. As observed, an asymmetric SC device incorporating NiCo‐LDH/NiOOH/ALD‐NiO and AC electrodes offered high energy density (72.6 W h kg^–1^) with superior cyclic ability after 10 000 cycles. Two ASC cells linked in series were used to effectively illuminate a white LED after charging at 3.2 V to demonstrate the merits of ALD assistance. Among the available pseudocapacitive materials, Ni(OH)_2_ containing mixed oxidative states and its hybrids with conducting materials have generated immense interest owing to their abundant availability, high theoretical capacity.^[^ ^286^, ^287^ ^]^ To this end, nickel‐based layered double hydroxides (LDHs) are extensively pursued, wherein additional trivalent metal ions replace the Ni ions and improves the cyclic stability because of the synergistic nature of the metal cations. To enhance the charge transport kinetics and avoid the aggregation of nanostructured active materials during CD cycling, Wang et al.^[^ ^287^ ^]^ combined the ALD and hydrothermal processes to develop a simple, tunable, and repeatable procedure that can synthesize hierarchical nanotubes assembled using NiAl LDH nanosheets.^[^ ^287^ ^]^ The authors employed a sacrificial ALD Al_2_O_3_ layer to synthesize the NiAl LDH. First, NiC_2_O_4_ nanowires were hydrothermally grown, and subsequently, Al_2_O_3_ was deposited via ALD to form core–shell NiC_2_O_4_@Al_2_O_3_ nanohybrids. The optimized (10 ALD cycles with Al_2_O_3_) NiAl LDH demonstrated large initial capacitance value of 2123.7 F g^–1^ at 0.5 A g^–1^, high rate, and cyclability (retaining ≈92% over 10 000 cycles at 5 A g^–1^). The high electroactivity of the NiAl‐LDH can be attributed to the optimal Ni^2+^/Al^3+^ ratio, in addition to the precise control of the nanotube diameter and nanosheet thickness by optimizing the number of ALD Al_2_O_3_ cycles.^[^ ^287^ ^]^ In another study, Wang et al.^[^ ^288^ ^]^ integrated ALD and hydrothermal techniques for in situ growth of hierarchical Ni(OH)_2_/NiAl‐LDH@C) onto NF. By utilizing the hierarchical nanostructure with ultrathin carbon shell, this Ni(OH)_2_/NiAl‐LDH@C electrode displayed an extremely high areal capacitance value of 14.0 F cm^–2^ at 5 mA cm^–2^, improved cycling performance (87.2% retention after 2000 cycles), and cycling stabilities similar to battery‐like SC electrode. Ternary NiCo_2_O_4_‐based materials recently garnered scholarly attention owing to its high specific capacitance, affordability, and environment affability. The superior electrochemistry of NiCo_2_O_4_ originates from the presence of both Ni and Co ions, which endow it with high electroactivity, redox reactivity, and electrical conductivity compared to the corresponding binary oxides (i.e., NiO and Co_3_O_4_).^[^ ^289^ ^]^ Alshareef et al.^[^ ^289^ ^]^ prepared a core–shell nickel cobaltite–titanium nitride (NiCo_2_O_4_@TiN) nanohybrid by coating a conformal layer of TiN on NiCo_2_O_4_ nanofiber array via ALD processing using TiCl_4_ and NH_3_ over hydrothermally prepared NiCo_2_O_4_ nanofiber arrays at 350 °C for 300 deposition cycles. In this nanohybrid, ALD‐TiN offered mechanical support and maintained its mechanical reliability over repeated cycles. Upon applying in all‐solid‐state symmetrical SCs, the core–shell structured NiCo_2_O_4_@TiN nanocomposite delivered a high mass power density (58.205 mW cm^−3^), corresponding to a moderate energy density (0.061 mW h cm^−3^) along with superior cyclic behavior (capacitance retention of ≈70% over 20 000 cycles).^[^ ^289^ ^]^

**Figure 14:**
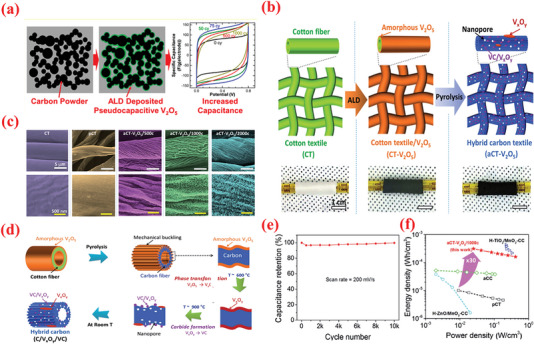
a) Schematic illustration of ALD‐coated carbon powder and corresponding cyclic voltammetry scans of prepared electrodes. Reproduced with permission.^[^ ^243^ ^]^ Copyright 2015, American Chemical Society. b) Schematic illustration of preparation procedure of hybrid carbon textile (aCT‐V_2_O_5_) from raw cotton textile (CT). Hybrid carbon textile was mechanically flexible and scrollable as observed in digital photos (bottom of panel (b)). c) SEM images of representative cotton and carbon textiles. d) Surface morphology and phase structure characteristics of textiles. Pyrolysis of V_2_O_5_‐coated cotton generated several wrinkles, nanopores, and nanoparticles on as‐obtained carbon textile. e) Cycling stability measured at a scan rate of 200 mV s^−1^ for 10 000 cycles. f) Ragone plot for symmetric supercapacitors composed of hybrid textile electrodes. Reproduced with permission.^[^ ^292^ ^]^ Copyright 2017, Wiley‐VCH.

To address volume expansion and conductivity challenges in active materials, novel electrode architectures such as core‐shell structures, capable of absorbing volume stress while maintaining high conductivity.^[^ ^290^ ^]^ Ren et al.^[^ ^240^ ^]^ demonstrated a unique ZnO NWs/Ni core/shell configuration for a pseudocapacitor electrode. First, the ZnO NWs core was hydrothermally obtained on Ti substrate using an ALD ZnO seed layer, followed by PEALD of a few layers of the Ni shell on ZnO NWs. During the ALD process, the presence of ZnO induced oxidation in certain deposited Ni layers, which resulted in the formation of NiO at the ZnO/Ni interface. The resulting Ti/ZnO NWs/Ni sample with a 30‐nm‐thick Ni–NiO shell layer delivered a high specific capacitance of ≈2440 F g^–1^, moderate rate (retained 80.5% at fast CD current rates) and cycling (retained 86.7% after 750 cycles at 10 A g^–1^) performances. Another core/shell nanoarray structure has been reported with ALD‐grown TiO_2_ NTs as its core and electrodeposited MnO_2_–C shells for high‐performance psuedocapacitive electrodes for SCs.^[^ ^291^ ^]^ Briefly, Co_2_(OH)_2_CO_3_ template array was initially prepared via hydrothermal technique, over which a thin coating of TiO_2_ was deposited by ALD processing of TiCl_4_ and H_2_O precursors. As a cathode material for asymmetric SCs, the TiO_2_/MnO_2_–C arrays exhibited a high specific capacitance (880 F g^–1^ at 2.5 A g^–1^) with suitable cyclic stability (retained 94.3% capacity over 20 000 cycles) because of its morphological advantage. PC scaffolds hosting TMOs and TMDCs have been explored to obtain simultaneous improvements in energy and power densities. In ref. [243], two commercially available AC materials with varying pore sizes and surface areas were processed with acid and covered with ALD‐V_2_O_5_ using VTIP and H_2_O at 150 °C.^[^ ^265^ ^]^ The mesoporous G60 increased its specific capacitance by 46% after 75 ALD‐V_2_O_5_ cycles and sustained through 10 000 CD cycles with 89% capacitance retention and efficiency (**Figure** **14**a). The carbothermic breakdown of V_2_O_5_ [C + V_2_O_5_ → C′ + VC + CO/CO_2_] induced chemical and mechanical stimulation of CC weave, which created a porous conductive substrate of large surface area. For instance, Lee et al.^[^ ^292^ ^]^ reported porous CC functionalized with both V_x_O_y_ and VC layers by ALD‐V_2_O_5_, followed by a facile pyrolysis process. Typically, the bare CC first coated with V_2_O_5_ via ALD utilized vanadium (V) oxytriisopropoxide and H_2_O at 150 °C to yield CT‐V_2_O_5_ (Figure 14b). Thereafter, the carbothermic conversion of V_2_O_5_ (V_2_O_5_ → V_x_O_y_ → VC) was performed by annealing CT‐V_2_O_5_ at 900 °C under vacuum. In particular, carbonization did not impact the bare CC or the large reduction in volume with slight variations in the morphology observed in Figure 14c. In contrast, heating in presence of VO_x_ film produced several nanoparticles, nanopores, and wrinkles on the CC threads. By using chemical etching and carbide production, the CC surface was scratched following the reaction of C + V_x_O_y_ → C′ + VC + CO/CO_2_(g). The authors graphically described the observed measures to understand the mechanism through which the wrinkles, nanopores, and nanoparticles were produced. The development model of the wrinkle/curls was adopted from the dehydration of fruits, according to which more wrinkles are produced with thinner oxides (Figure 14d). Thus, the as‐obtained C/V_x_O_y_/VC composite electrode demonstrated appropriate synergistic effects of the EDLC and PC behavior, which increased the specific capacitance (≈514 mF cm^–2^ (or 80 F g^–1^)) and long‐term cycling stability of the optimized electrode after 10,000 CD tests (Figure 14e,f). The high capacitive performances can be attributed to the mutual supporting nature of both VC and V_x_O_y_ in the composite, such that VC activated the textile and V_x_O_y_ enhanced the electrochemical performance by creating numerous subnanopores on carbon that suitably enhanced the double‐layer formation.^[^ ^292^ ^]^

### 3.4 Protective/Passive Coatings

The active electrode is a key factor for determining the performance of SCs because electrical energy is recovered through charge separation in a Helmholtz double layer.^[^ ^293^ ^]^ To realize high‐performance SCs, especially those operating at high voltages, minimizing current leakage is a crucial challenge because it grows exponentially with imposed current. In contrast to the respective masses of the two electrodes, the operational voltage may increase over 2.7 V.^[^ ^293^, ^294^ ^]^ Recent studies^[^ ^206^, ^295^, ^296^, ^297^, ^298^, ^299^ ^]^ have attempted to improve the cell voltage of AC‐based EDLCs via ALD‐assisted stabilization of the electrolyte and electroactive surface functionalities at the porous carbon surface. Scientists investigating in the area of energy storage are inspired by this ingenious application of dielectrics as insulating layers. In EDLC cells with organic electrolytes, the commercially available AC material displayed a operational voltage of ≈3.5 V with an ultrathin Al_2_O_3_ prepared by ALD. The ultrathin oxide layer successfully ceased either the dissolution of the active materials or the direct contact of the electrolyte with the active sites on AC, thereby inhibiting the breakdown of the electrolyte throughout the cyclic operation. The Al_2_O_3_ modification enabled the graphene supercapacitor cells to achieve 4.4 V by employing mesoporous graphene sponge.^[^ ^295^, ^296^ ^]^ These findings triggered a deeper interest in researching the AC‐based EDLC device's route to its higher voltage capability. As the self‐discharge phenomenon crucially impacts the production of current EDLCs, it must be suppressed along with the high‐voltage CD weakening problem. In this regard, MOs (e.g., Al_2_O_3_) are gaining increasing attention for passivating the electrode surface of AC, and yield a cell voltage above 3 V. For instance, Tan et al.^[^ ^300^ ^]^ reported a cell voltage of ≈3.5 V in EDLCs containing organic electrolytes by passivating the AC surface with an ALD grown ultrathin (1–2 nm thick) Al_2_O_3_ layer, which effectively avoided the dissolution of active materials as well as electrolyte decomposition by preventing direct contact between the electrode and electrolyte during the cycling tests. Notably, it offered higher cell voltages because of the thickened Stern layer.^[^ ^300^ ^]^ To enhance the self‐discharge characteristics of AC‐based EDLCs, the same scholars^[^ ^301^ ^]^ further demonstrated that the optimized number of 20 ALD cycles of Al_2_O_3_ over AC electrodes can enhance the cell voltage to 4 V and delivered 84% capacitance retention after 1 000 cycles compared to the 48% retention of pure AC cells.

**Figure 15:**
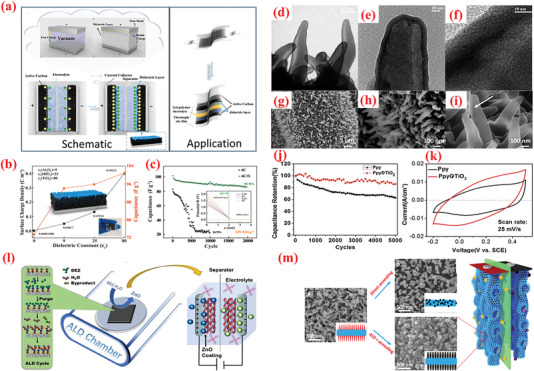
a) schematic illustration of the development of higher energy density EDLCs, as inspired by the evolution of a parallel‐plate capacitor with a microscale dielectric layer from a simple parallel‐plate capacitor. b) Flexible EDLCs, assembled by ALD modified electrodes. The surface charge density and specific capacitance of electrodes modified with multiple dielectric layers at 2.5 V. c) Cycle stability of AC‐Ti versus pristine AC at a cell potential of 3.5 V and current density of 0.5 A g^–1^. Reproduced with permission.^[^ ^302^ ^]^ Copyright 2021, Elsevier. (d–f) TEM characterization of hollow core–shell ppyʘTiO_2_ nanoarrays. g–i) SEM images of hollow core–shell ppyʘTiO_2_ nanoarrays on carbon cloth. j) Cycling stability test and (k) CV curves of ppy nanowires and hollow core–shell ppyʘTiO_2_ nanoarrays. Reproduced with permission.^[^ ^303^ ^]^ Copyright 2018, Elsevier. The figure displays a schematic of ALD coating and an EDLC cell modified with crystalline ZnO in (l). Reproduced with permission.^[^ ^304^ ^]^ Copyright 2021, MDPI. m) Controlled transformation of FeOOH NRs to Fe_2_N for ASCs device. SEM images of FeOOH NRs and NPs developed on carbon fiber followed by ammonia annealing, Fe_2_N–Ti_2_N C‐S NRs found via ALD‐TiO_2_ on FeOOH NRs followed by ammonia annealing. Reproduced with permission.^[^ ^253^ ^]^ Copyright 2016, Elsevier.

To eliminate and suppress the reactivity of various surface functionalities and enhance the capacity of the AC electrodes, Zhang et al.^[^ ^294^ ^]^ recently introduced a combination of oxygen plasma treatment and ALD–Al_2_O_3_ growth on AC electrodes and deeply investigated the effects of both these treatments on electrode performance. The nanometer‐thick (≈2 nm) oxide coating over oxygen‐containing AC electrodes effectively promoted the functionalities of C–O but suppressed those of the C═O and O–C═O groups, thus reducing the activity/reactivity through the prolonged cyclic operation. Consequently, the novel strategy of initially creating more defects on AC surface via oxygen doping, followed by ALD passivation resulted in excellent rate performance (40.6 F g^–1^ at 5 mA cm^–2^ and 20.1 F g^–1^ at 100 mA cm^–2^) and cycling stabilities (retained ≈90% after 5 000 CD cycles) at 3.2 V, which was > 6% higher than pure and > 30% higher than plasma‐treated AC electrodes. In addition, the electrodes treated with both plasma and ALD, displayed almost no surface alterations after cycling tests. Thus, the combination of the plasma treatment and ALD coating yielded a stable cycling performance of the AC‐based SCs. Recently, Han et al.^[^ ^302^ ^]^ compared the electrochemical performance of the AC electrodes modified via ALD with various dielectric layers, e.g., Al_2_O_3_, HfO_2_, and TiO_2_. The electrode modified with ALD–TiO_2_ exhibited a significant increase of 35% in specific capacitance compared with pristine AC electrodes. Furthermore, the ALD modification enhanced the operation voltage up to 3.5 V along with remarkably high capacitance retention (83% over 20 000 cycles at 0.5 A g^–1^) (**Figure** **15**a–c).^[^ ^302^ ^]^

Without binders or other additions, the direct development of conducting polymer nanoarrays on current collectors. Such materials are well‐connected to the current collector via mechanical and electrical means. Another tactic is to develop the composite electrode by combining the conducting polymers with carbonaceous or TMO components. As reported, a number of hybrid materials link the advantages of the synergistic effect from individual materials. Yang et al.^[^ ^303^ ^]^ created and constructed a hollow shell on polypyrrole nanowires (ppy) using ALD. Initially, ppy was electrodeposited onto flexible CC fabric, after which a uniformly thin ZnO sacrificial layer and a thin TiO_2_ as a shell were coated on the surface of the nanowires, as depicted in Figure 15d–i). After removing the ZnO layer, a nanogap formed between the ppy core and TiO_2_ shell, creating a hollow core–shell ppy‐TiO_2_ arrays. The conformal ALD‐TiO_2_ as a shell can aid in shielding the active material from mechanical degradation over continued CD tests, which significantly improves the cycling constancy (Figure 15j,k).^[^ ^303^ ^]^

Generally, AC exhibits instability above a certain threshold potential range, which restricts their use to lower operating voltages. In a recent study, Wu et al.^[^ ^304^ ^]^ deposited ZnO via ALD to enhance the cycling performance of AC‐based SC electrodes, as mentioned in Figure 15l. To achieve high electric conductivity and strong capacitance retention among the AC materials, the authors optimized the ZnO coating process by altering the precursor exposure period, number of ALD cycles, and growth temperature. A longer pulse duration (400 ms) of precursors distinctly resulted in the optimal formation of crystalline ZnO phase, which consequently enhanced the electrochemical performance. The electrode obtained after 20 ALD cycles of ZnO at 70 °C using TEABF4/acetonitrile electrolyte exhibited a capacitance value of 23.13 F g^–1^ at 5 mA cm^–2^ in addition to an increased capacity retention (50%) at 3.2 V (exceeding the working potential of 2.7 V for a conventional EDLC device) and long cyclic stability of 5000 cycles.^[^ ^304^ ^]^ In addition to iron oxide, Fe_2_N can be considered a suitable electrode for SCs as its capacitance is within the desired range. Accordingly, Fe_2_N–Ti_2_N nanorod electrodes have been used in 2.0 V quasi‐solid‐state symmetric capacitive device described by Zhu et al.^[^ ^253^ ^]^ Fe_2_N is obtained from its oxyhydroxide precursor and surface‐protected by an ultrathin and stable Ti_2_N shell, which prevents the formation of the original nanorod structure (Figure 15m). The symmetric device enables extremely high scan rates up to 50 V s^–1^ and provides a reasonably constant capacitance during CD cycling (82 F g^–1^ with 99% capacitance retention after 20 000 cycles) owing to the advantageous characteristics of unique structural properties.

With larger ions such as those contained in the electrolyte or the additional layer of dielectric over the C surface, the Stern layer thickness can be increased to permit a higher potential of 3 V. Tan et al.^[^ ^300^ ^]^ studied the ALD‐modified AC electrodes to discuss the process–structure–property relations. In total, four distinct types of dielectric materials were used in the experiments, and the authors fabricated insulating Al_2_O_3_ with high‐*k* dielectrics, mixed Zn–Al_2_O_3_ with moderately high dielectrics, high dielectric permittivity TiO_2_, and conductive magnetic Mn–CoO_x_. Regardless of the electronic nature of the ALD produced oxides, coin‐cell SCs exhibit a spectacular increase in capacitance retention over 1000 cycling performances. As discovered, ALD–oxides prepared with 20 ALD cycles (or thickness: 1–2 nm) efficiently produced dependable CD process, reduced impedance, and remarkably small current leakage. As straight impacts mass transfer, the creation of EDLC, and e^−^transfer at the interface, the wettability of the active material surfaces acts a significant character in several interfacial activities. Although the aligned integration of the CNT on the chip is favorable for a microscale supercapacitor, controlling the wettability and microstructure is a more challenging problem. In a study, Avasthi et al.^[^ ^305^ ^]^ coated a VACNT sample with 3‐nm‐thickness ALD–TiO_2_, rendering the electrode superhydrophilic. Compared to naked VACNT in KOH, the created VACNT–TiO_2_ composite exhibits over 100 times higher energy density, 20 times higher capacitance, 13 times higher power density, with improved capacitance retention. Combined with electrolyte engineering, this straightforward hybrid technique can improve the electrochemical performance in various applications requiring wide adjustments of the wettability criterion.

**Figure 16:**
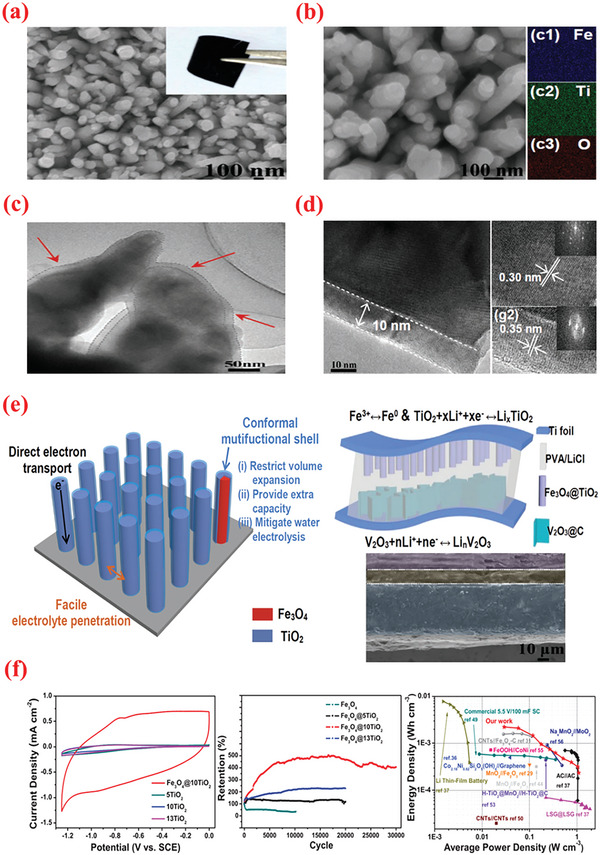
a,b) FE‐SEM pictures of Fe_3_O_4_@10TiO_2_ nanorod arrays (NAs). Elemental mapping marks the presence of Fe, Ti, and O elements. c) TEM images of mixture NAs, displaying the occurrence of TiO_2_ shell. d) HRTEM images of g1) Fe_3_O_4_ core and g2) TiO_2_ shell. e) Graphic design of structural characteristic of Fe_3_O_4_@ALD TiO_2_ NAs as electrode for the test. Visual representation of V_2_O_3_@C//Fe_3_O_4_@TiO_2_ quasi‐solid‐state SC along with X‐SEM image. f) CV curves, cycling performance, and Ragone plot comparison of Fe_3_O_4_@TiO_2_ electrodes and ALD grown TiO_2_ directly on Ti substrate. Reproduced with permission.^[^ ^306^ ^]^ Copyright 2018, Wiley‐VCH.

Although the enormous capacity of FeO_x_ is promising for electrode applications, its cycle instability causes one of the most severe issues. Li et al.^[^ ^306^ ^]^ employed a TiO_2_ with conformal coating via ALD to enable the solid protection on Fe_2_O_3_ in neutral‐pH 1 M Li_2_SO_4_ for the first time. Fe_3_O_4_@TiO_2_ hybrid were obtained by growing FeOOH NRs vertically on the Ti foil through hydrothermal process and ALD–TiO_2_ process adopted with TiCl_4_ and H_2_O precursors. The resulting sample analyzed by SEM and TEM images confirmed that all the samples were grown with abundant vertically aligned NRs, as portrayed in **Figure** **16**a–e. Distinctive TEM results further displayed that the TiO_2_ layers entirely covered the individual NRs surface with uniform thickness with several lattice fringes (Figure 16d). The Fe_3_O_4_@TiO_2_ hybrids ensues numerous essential advantages, expressed in Figure 16e as ALD ensures excellent Fe_3_O_4_ adhesion, uniformly high TiO_2_ shell uniformity, and accurate control of the shell thickness along with Li^+^ insertion into TiO_2_ with negligible volume variations, which facilitates the stable and efficient reduction in volume fluctuations of the underlying Fe_3_O_4_ nanorod during test. The decline in the ongoing capacity of Fe_3_O_4_ was ceased by an optimized TiO_2_ shell of 10 nm, which exhibited an optimal cyclic behavior of 30 000 cycles and high energy/power density (Figure 16f).^[^ ^306^ ^]^

**Figure 17:**
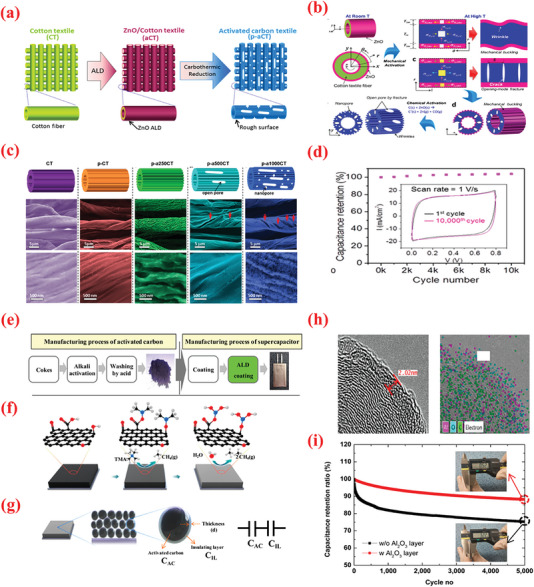
This figure depicts the fabrication of activated carbon textile via carbothermic reduction. The schematic in a) illustrates the preparation procedure for activating carbon from cotton using carbothermic reduction, while b) shows the resulting surface roughening due to mechanical buckling and fracture during pyrolysis with ZnO. The extent of buckling and fracturing varied with ZnO thickness, resulting in increased surface area and pore volumes of carbon textiles. The carbon fiber underwent both mechanical and chemical activation. The proposed mechanism for wrinkle and crack formation during carbothermic reduction is shown in c), where the cotton/ZnO fiber is visualized as a cylinder‐shaped fiber with two layers. Two elemental sections were defined based on cylindrical symmetry to explain the origin of wrinkle and crack formation. The resulting carbon textile showed wrinkles and cracks. The cycling stability of the carbon textile was measured at a scan rate of 1 V s^−1^ for 10,000 cycles and presented in d). Reproduced with permission.^[^ ^279^ ^]^ Copyright 2016, American Chemical Society. e) A depiction of the fabrication procedure used to create a supercapacitor with Al_2_O_3_ deposition on activated carbon. Schematic illustrations of the chemical reaction involved in the ALD‐Al_2_O_3_ coating process on activated carbon are shown in f) and g). The HRTEM and mapping image of activated carbon coated with ALD‐Al_2_O_3_ is displayed in h). The figure also shows the capacity retention of the supercapacitor during 5000 cycles at 70 °C in i), with the thickness after 5000 cycles displayed in the insets. Reproduced with permission.^[^ ^295^ ^]^ Copyright 2015, American Chemical Society.

Carbonization creates conductive, porous constituents for improved electrode properties, but achieving dense porosities is challenging. Chemical activation using multiple steps to create active sites is limited. Electrode preparation with additives and binders affects conductivity and storage capability. Thus, essential to develop etchant‐free activation techniques for high‐performance electrodes. As depicted in in **Figure** **17**a, Lam et al.^[^ ^279^ ^]^ introduced a method to active the CC for high SCs using MO sacrificial mediator for carbothermic reduction. In this process, ALD–ZnO films with uniform and conformally coated CC and further thermal annealed to fade Zn and O species by following reaction^[^ ^279^ ^]^

(20) $$\left(C\left(s\right)+\mathrm{ZnO}\left(s\right)\to C^{\prime }\left(s\right)+\mathrm{Zn}\left(g\right)+\mathrm{CO}\left(g\right)\right)$$

The variations in thermal stress that increased the surface area caused mechanical buckling and opening mode fracture in 2D materials in Figure 17a,b. The SEM images revealed the distinct morphological variances on the CC surface after carbothermal process, thereby signifying the influence of the ZnO layer in causing these morphological alterations. The authors expected a single CC yarn covered with ZnO layer as a cylinder shielded with an even ZnO film. Based on the cylindrical symmetry, we established two elemental sections, the first in the *r*‐plane and the second in the *r*–*z* plane (Figure 17c). The carbothermic reduction‐produced CC surface displayed remarkable synergistic capabilities of high power/densities with a cyclic performance 20 times greater than that of non ZnO–CC (Figure 17d).

Operating voltage enhancement is an effective method for SC with high energy density. The electrochemical activity of commonly applied AC‐based electrodes typically degrades beyond 2.5 V. Hong et al.^[^ ^295^ ^]^ presented an ALD‐coated ACs with ultrathin Al_2_O_3_ film (thickness: 2 nm) for SC electrodes as ALD provides control over nanolayer thickness. The ultrathin Al_2_O_3_ layer–ALD technique is schematically represented in Figure 17e–h. Primarily, on the AC surface, the TMA is dosed on the densely hydroxyl groups generated during acid cleaning. The TMA fragments react with the –OH clusters forming C−O−Al(CH_3_)_2_ on the surface. AL–OH moieties were generated by the reaction between water and AlCH_3_ molecules after releasing H_2_O. The activated surface with –OH groups generated during acid cleaning was caused by functionalization of the surface sites for the ALD reaction. The prepared electrodes exhibited a 39% increase in energy density at an operating voltage of 2.5 V and an outstanding stability at 3 V, corresponding to ≈74% retention of the original potential until 50 h after complete operation and adequate retention with 88% after 5 000 cycles, which resembled to 31 and 17% enhancements from plain AC, respectively (Figure 17i). This ALD‐engineered surface modification affords a facile approach of improving the electrochemical activity, especially for the carbonaceous electrodes across a wide range of applications. Pint et al.^[^ ^307^ ^]^ demonstrated the construction of a solid‐state dielectric capacitor using free‐standing VA‐SWNTs framework yielding a comparable performance to SCs. The authors claimed that the performance of this energy storage architecture is consistent with the theoretical predictions using ALD as a method to uniformly cover the VA‐SWNTs substrate with Al_2_O_3_, and the Al–ZnO films will act as counter‐electrodes. The estimated capacitance for a 15‐nm‐thick Al_2_O_3_ dielectric layer was 11 mF cm^−3^, which is proximate to the experimentally measured value of 23 mF cm.^−2[^ ^307^ ^]^

### 3.5 Integration with Silicon Technology

**Figure 18:**
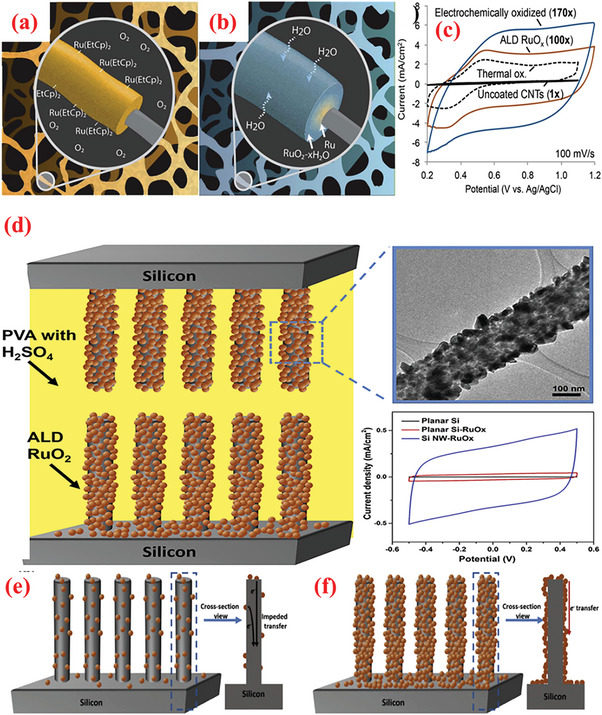
Pictorial explanation and representation of ALD‐RuO_x_ as electrodes for SCs. a) Schematic illustration of uniform and conformal ALD deposition of RuO_x_ on a large‐surface‐area porous electrode. Consecutive dosing of Ru(EtCp)_2_ and oxygen enables layer‐by‐layer deposition of RuO_x_ thin films. b) Pictorial explanation and practical demonstration of how pseudocapacitance can be improved by post‐ALD electrochemical oxidation. It also highlights the chemical activation process that is utilized to create a hydrated RuO_x_ SC electrode that exhibits high proton conductivity. c) CV measurements of CNT and porous Si ALD RuOx electrodes. Reproduced with permission.^[^ ^311^ ^]^ Copyright 2015, Royal Society of Chemistry. d) Schematic of symmetric solid‐state on‐chip supercapacitors based on SiNW–RuO_x_ electrodes. TEM images of SiNW1‐150cyc sample. Comparison CVs of prepared samples. Schematics of charge transfer in e) 150cyc electrodes, and f) 400cyc electrodes. Reproduced with permission.^[^ ^312^ ^]^ Copyright 2017, Elsevier.

The incorporation of energy‐storage devices with on‐chip electronic circuitry is highly desirable to satisfy the continually increasing demand of device miniaturization, which requires local energy delivery.^[^ ^105^, ^114^, ^308^ ^]^ In this regard, research should focus to develop a synthesis approach that is compatible with the present Si‐based CMOS technology.^[^ ^309^ ^]^ Among various candidates, RuO_x_ is a promising pseudocapacitive material owing to its high gravimetric capacity (≈1000 F g^–1^), high conductivity, and adequate cyclability.^[^ ^310^ ^]^ Recently, RuO_x_ has gained popularity in micro‐SCs owing to the requirement of less material. However, the ALD growth of RuO_x_ involves the following challenges: i) difficulty to control the oxidation of Ru, with products containing mixed phases from metallic Ru to RuO_2_, and ii) ALD deposition at higher temperatures (>270 °C) inhibits the formation of hydrated oxide. To overcome these challenges, Lin et al.^[^ ^311^ ^]^ combined low‐temperature ALD with a post‐ALD electrochemical oxidation process to develop advance Si‐based micro‐SCs. The ALD process was conducted using bis(ethylcyclopentadienyl)ruthenium (II) (Ru(EtCp)_2_) and O_2_ at varying temperatures from 270 to 400 °C (**Figure** **18**a–c). The CV plots of ALD films grown at various temperatures display higher capacitance values for films deposited at lower temperature (e.g., 270 °C) in comparison to that grown at higher temperatures (e.g., 350 and 400 °C). Such a behavior can be attributed to the temperature‐induced dehydration of the MO films, wherein higher temperatures yield greater dehydration that consequently results in lower proton conductivity and lower capacitance values compared to the films deposited at lower growth temperatures.^[^ ^311^ ^]^ Compared to bare CNT, the products obtained with ALD deposition and electrochemical oxidation displayed 100‐ and 170‐times higher capacitances, respectively. Moreover, the obtained specific capacitance value (644 F g^–1^) approximated the theoretical limit of RuO_x_ at a power density of 17 kW kg^–1^. The CD tests conducted at ultrafast scan rates of 20 V s^–1^ revealed a 17% increase in capacitance values over 10 000 cycles.^[^ ^311^ ^]^ In another attempt of harnessing the excellent properties of RuO_x_, Thompson et al.^[^ ^312^ ^]^ fabricated solid‐state on‐chip SCs from RuO_x_‐coated Si NWs using a CMOS compatible method. The ability of ALD to yield high‐precision thin‐film deposition even on high‐aspect‐ratio structures produced a uniform coating of RuO_x_ over Si NWs, which displayed considerable enhancements in the as‐developed devices (Figure 18d–f). The electrochemical characteristics of the coated NWs were assessed using neutral‐pH Na_2_SO_4_ electrolyte, exhibiting a specific capacitance of 19 mF cm^–2^ at 5 mV s^–1^, which was approximately two magnitudes higher than that obtained without RuO_x_ coating. Finally, solid‐state symmetric on‐chip SCs were fabricated using RuO_x_‐coated NW arrays as electrodes and solid polymer electrolyte, exhibiting a high specific capacitance of 6.5 mF cm^–2^ at 2 mV s^–1^, retaining 92% of the initial capacitance over 10 000 cycles at 0.4 mA cm^–2^.^[^ ^312^ ^]^

**Figure 19:**
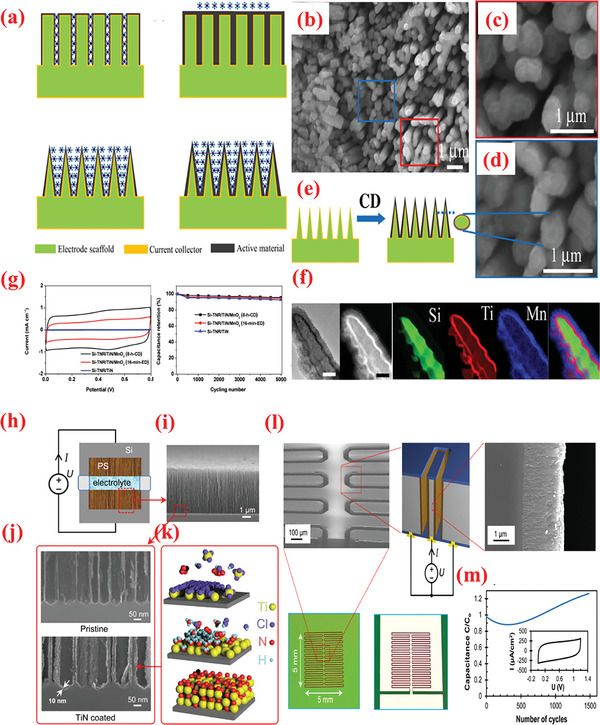
a) Schematic representation of electrode design. b) Top view of SEM images of Si‐TNR/TiN (8‐h‐CD) sample after ultrasonic treatment, c) Tips of Si/TiN/MnO_2_ nanorods recorded from area marked in b) with red rectangle, d) Cleaved profile of Si/TiN/MnO_2_ nanorods recorded from area marked in b) with blue rectangle. e) Illustration of CD coating of MnO_2_ on Si‐TNR/TiN scaffold. f) TEM images, high‐angle annular dark‐field STEM images, and corresponding elemental maps of a nanorod tip. g) Comparison of Si‐TNR/TiN/MnO_2_ (8‐h‐CD) electrode, Si‐TNR/TiN/MnO_2_ (16‐min‐ED) electrode, and Si‐TNR/TiN electrode of CV curves recorded at 100 mV s^−1^, and cycling performance at 100 mV s^−1^. Reproduced with permission.^[^ ^313^ ^]^ Copyright 2017, Elsevier. h) Pictorial description of fabrication processes of PS–TiN SC with distinct electrode chips. (i–j) cross‐section SEM images of porous Si layer and conformally coating of TiN on PS layer. k) Schematic diagram of ALD grown TiN thin films. l) An oblique SEM image of the trenches that divide the electrodes in a cross‐sectional schematic of a device. The electrodes are coated with a TiN layer and have aluminum contact pads on the rear. m) CV profiles at 100 mV s^−1^ (inset) and capacitance retention. Reproduced with permission.^[^ ^314^ ^]^ Copyright 2016, Elsevier.

Because of the high energy density, on‐chip microscale batteries are promising candidates for managing the sleep‐mode function of the sensor networks and IoT essentials. In this regard, Lu et al.^[^ ^313^ ^]^ prepared a ternary composite of Si/TiN/MnO_2_ tapered nanorod arrays to function as a high‐specific‐capacitance electrode for on‐chip SC applications. The various components of the composite were prepared as follows: silicon with cyclic deep reactive ion etching, TiN via ALD, and MnO_2_ via a solution‐based chemical deposition. A schematic illustrating the structural advantages of these tapered nanorod arrays for SC applications is displayed in **Figure** **19**a–g. To completely utilize the structural advantages, both the current collector and active material must be conformally deposited. Based on these merits, the Si‐TNR/TiN/MnO_2_ composite prepared as an electrode provided an areal capacitance of 81.6 mF cm^–2^ at 5 mV s^–1^ (preserved 41.3 mF cm^–2^ at 200 mV s^–1^), which was 70 times higher than that observed for the SiTNR/TiN electrode. The results successfully demonstrated the immense potential of novel tapered nanorod array as electrode scaffolds for designing high‐performance on‐chip electrodes. Porous Si (PSi)‐based nanostructures are ideal for integrating energy storage with active devices, supporting the focus on autonomous sensor networks, wearable electronic devices, and portable device miniaturization. For instance, Grigoras et al.^[^ ^314^ ^]^ demonstrated the production of highly conductive, wettable, stable SC electrodes by conformally coating an ultrathin TiN film on PS (Figure 19h–m). The SCs could adequately perform in aqueous as well as organic electrolytes. The realization of a conformal coverage of PSi persists as a challenge owing to the extremely high aspect ratios (up to 1:1000 or above). Nevertheless, ALD is a versatile technique that can be optimized for features of extremely high aspect ratio. The authors deposited a ≈10‐nm‐thick TiN layer inside the PSi matrix via an optimized ALD process, which enabled the fabrication of highly efficient SC electrodes with nearly ideal EDLC‐type characteristics. The resulting electrode offered a higher specific capacitance of 15 F cm^–3^, energy density of 1.3 mW h cm^–3^, power density of 214 W cm^–3^, and exceptional cycling test (> 13 000 cycles). This form of in‐chip supercapacitor establishes an avenue to new kinds of miniature components and assemblies that require on‐site integrated energy storage.

A few recent studies demonstrated the use of ALD coatings for producing 3D micro‐SCs. For instance, Brown et al.^[^ ^315^ ^]^ fabricated a full‐cell 3D micro‐SC with a potential of rapid and scalable manufacturing. As depicted in ref. [315], the authors employed ALD to deposit both TiO_2_ and TiN on CVD‐prepared VACNTs to produce symmetrical TiN‐TiO_2_‐VACNT 3D MSC SCs. The electrochemical results displayed a 74‐fold enhancement of the optimized electrode in terms of specific capacitance (5.18 mF cm^–2^) compared to pure VACNT based MSCs (0.07 mF cm^–2^). The improved performance of TiN–TiO_2_ composite compared to only TiO_2_ or TiN was attributed to the nitrogen doping of TiO_2_ (i.e., titanium oxynitride or TiON) in presence of TiN. The authors demonstrated that an ALD deposition with TiN/TiO_2_ ratio of 1:1 enabled the optimum electrode performance and MSC operation, with the best performance of MSCs. For instance, although the TiN(300)‐TiO_2_(100)‐VACNT exhibited the highest capacitance (11.17 mF cm^–2^), energy (5.474 mW h cm^–2^), and power density (5.469 mW cm^–2^) at 0.1 mA cm^–2^ owing to the thicker TiN film, this MSC was nonfunctional.^[^ ^315^ ^]^

**Figure 20:**
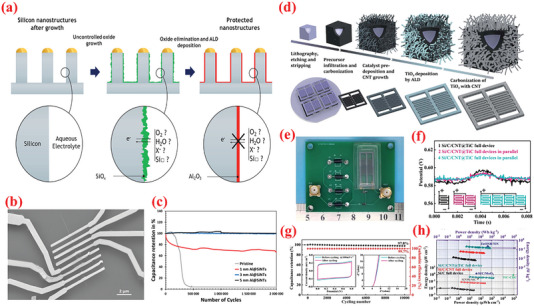
a) Replacement of SiO_2_ native oxide of silicon nanostructure by nanometric layer of high‐*k* dielectric alumina. b) SEM images of electrical contacts on a 5‐nm‐thick alumina capped on Si NWs. c) Cycling performance of SiNTs microsupercapacitor devices at 0.5 mA cm^−2^. Reproduced with permission.^[^ ^316^ ^]^ Copyright 2022, Elsevier. d) Schematics of synthesis procedure of 3D interdigitated Si/C/CNT@TiC MSC involving bulk micromachining, photoresist pyrolysis, CVD, ALD, and in situ thermal annealing. e) Optical image of full‐wave bridge rectifier circuit. f) Low‐pass filtering circuit and on‐chip application based on 3D MSCs. Output of circuit with Si/C/CNT@TiC full device connected in parallel. g) Long‐time cycling performance (CV curves and Nyquist plots before and after cycling in inset). h) Ragone plots of three devices. Reproduced with permission.^[^ ^317^ ^]^ Copyright 2022, Royal Society of Chemistry.

Apart from the requirements in terms of operating conditions, the integration of devices with silicon microelectronics technology is an important consideration toward miniaturization and IoT.^[^ ^308^ ^]^ Therefore, compatibility between the fabrication methods and the techniques employed in wafer bonding technology is crucial for 3D integration. Vazquez‐Arce et al.^[^ ^316^ ^]^ demonstrated a thin‐film symmetric SC fabricated using sputtered Ru electrodes and ALD‐deposited Yttria‐stabilized Zirconia (YSZ) electrolyte, operating across a range of temperatures. The ALD deposition of YSZ electrolyte was performed using tetrakis(ethylmethylamino) zirconium (TEMAZr), and tris(methylcyclopentadienyl) yttrium (Y(MeCp)_3_) as the metal precursors for ZrO_2_ and Y_2_O_3_ at a temperature of 250 °C, and ZrO_2_:Y2O_3_ cycle ratio of 4:1. For an operating voltage of 1.8 V, the energy density increased with the temperature from 50 to 200 °C, for all tested values of thickness. The YSZ with a thickness of 45 nm exhibited the highest energy density values of 0.129 mW h cm^–3^ at 50 °C and 60.4 mW h cm^–3^ at 50 and 200 °C, respectively. The results obtained here are crucial for shifting from microscale to nanoscale wearable devices. The thickness‐dependent tunneling electrical conduction of an atomic‐level uniform Al_2_O_3_ over SiNWs is demonstrated using microelectronic measuring methods and nanolayer regulation of dielectric thickness via ALD. This finding sheds new light on this category of energy‐related materials and opens up novel avenues for creating scientific strides. As depicted in **Figure** **20**a,b, highly *n*‐doped SiNWs were coated with Al_2_O_3_ utilizing trimethylaluminum and H_2_O (0.06 s of TMA, 8 s of purging, 0.06 s of H_2_O, and 8 s of purging) until the appropriate thickness was achieved.^[^ ^293^ ^]^ Using tunneling current in an aqueous electrolyte, a Si‐based micro‐SC preserved with 3‐nm‐thick dielectric of Al_2_O_3_ demonstrated EDLC—first such instance for this material. In addition, it manifested an exceptional long‐term capacity, while retaining 99% capacitance over 2 million cycles. Such a technology, if applied to other energy materials, can realize significant advancements (Figure 20c).

Recently, Wang et al.^[^ ^317^ ^]^ fabricated a vastly conducting carbon film on etched Si template via bulk micromachining technique and carbonization for use as complex 3D framework electrode. Subsequently, CNTs and TiC nanotube (CNT@TiC) were consecutively synthesized via sputtering, CVD, ALD, and annealing process, resulting in a Si/C/CNT@TiC electrodes (Figure 20d–h) exhibiting high electrical conductivity and enhanced electrochemical performance with increasing reduction period. The symmetric MSC comprising Si/C/CNT@TiC electrodes presented a higher specific capacitance (7.42 mF cm^–2^ and 3.71 F g^–1^) at 5 mV s^–1^. Furthermore, full solid‐state MSC assembled with cold glass and PVA/LiCl electrolyte delivered a high energy and power density with ≈98% retention over 10 000 cycles.^[^ ^317^ ^]^

### 3.6 Current Collectors and Insulating Layers

**Figure 21:**
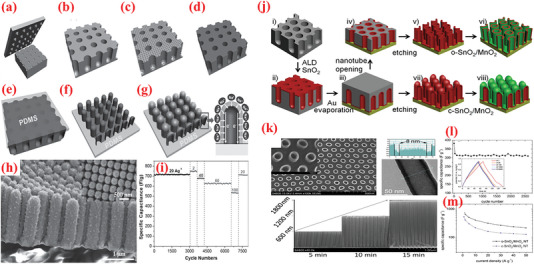
a–g) Schematic illustration of fabrication process of Pt and Pt/MnO_2_ NT arrays: a) Ni nanopillar stamp used for surface imprinting on an aluminum foil; b) preparation of alumina template (AT) through anodization followed by chemical etching; c) formation of dispersed Pt NPs on the template after a few cycles of ALD growth; d) formation of a continuous Pt NTAs after additional ALD growth cycles; e) AT covered with a mixed PDMS solution; f) removal of the AT to obtain a Pt NTAs on the PDMS substrate. g) electrodeposition of MnO_2_ to form Pt/MnO_2_ NT array for supercapacitor electrode. h) Tilted and top SEM images of MnO_2_ shell on Pt NTs after deposition of 90 s. i) Cycling stability of 30‐s‐NT electrode at random current densities up to 8000 cycles. Reproduced with permission from ref. [319] Copyright 2014, Wiley‐VCH. j) Graphical depiction of open‐end o‐SnO_2_/MnO_2_ NTAs and closed‐end *c*‐SnO_2_/MnO_2_ NTAs. k) SEM image of *o*‐SnO_2_ NTAs. X‐SEM image showing the dimension of NTs in reliance of template anodization period. TEM image confirming the hollow structure of NTs. l) Cyclic stability test at 5 A g^–1^ comprising C/D profile in inset. m) Rate capability of *o*‐SnO_2_/MnO_2_ and *c*‐SnO_2_/MnO_2_ NTAs. Reproduced with permission.^[^ ^239^ ^]^ Copyright 2014, Elsevier.

Leveraging the possibility of conformal growth in a highly controlled manner, ALD has been employed for fabricating microelectrodes.^[^ ^264^, ^275^, ^318^ ^]^ For instance, Lei et al.^[^ ^319^ ^]^ employed ALD to realize precise Pt NT inside nanoporous AAO template using Pt(MeCp)Me_3_ and O_2_ as precursors at a deposition temperature of 300 °C to yield current collectors for high‐performance SCs. The authors performed detailed experiments to examine the effects of nitrogen amount and Pt precursor pulsing period on Pt infiltration depth. Furthermore, the authors demonstrated the use of Pt nanotube arrays as high‐performance current collector for SCs by preparing core/shell Pt/MnO_2_ nanotube array via electrodeposition of MnO_2_ nanotubes on Pt cores (**Figure** **21**a–h).^[^ ^319^ ^]^ Consequently, this electrode presented high gravimetric/areal capacitances (810 F g^–1^ and 75 mF cm^–2^ at 5 mV s^–1^) including a high‐rate performance (68% retention) (Figure 21i). Furthermore, the cycling tests for random CD current rates from 2 to 100 A g^–1^ revealed almost no capacitance loss over 8000 cycles. The approach reported in this study enabled the practical utilization of Pt nanostructures in device miniaturization, e.g., current collectors.^[^ ^319^ ^]^

MnO_2_ offers multiple advantages for use in energy storage systems including high theoretical capacity, low cost, environment benevolence, and resource abundance.^[^ ^241^, ^320^, ^321^, ^322^ ^]^ In addition to these advantages, MnO_2_ suffers from limited electronic conductivity that affects the rapid movement of ions moving and weakens the structural strength, especially at the nanoscale. These limitations can be overcome by combining MnO_2_ with materials such as TiN that offer excellent electrical conductivity (≈5 × 10^4^ S cm^–1^ for the bulk material) for rapid electron transport as well as mechanical strength. In this regard, certain scholars^[^ ^323^ ^]^ reported the synthesis of MnO_2_ TiN NTs as efficient an electrode material. Because of the unique design maintaining close contact of MnO_2_ with both the interior and exterior surface of the TiN NTs, the prepared MnO_2_/TiN nanotubes yield a high value of capacitance (834 F g^–1^ at 9 A g^–1^) for use as SCs electrode. The combination of MnO_2_ and TiN into a single electrode system holds immense promise for fabricating electrode materials for SC devices^[^ ^239^ ^]^; however, it encounters two critical challenges: (a) high‐capacity materials such as MnO_2_ display a high resistivity (≈102–106 Ω cm^–1^) that severely compromises the performance of the overall device; (b) optimum configuration that consequently affords greater control over all structural parameters. In this perspective, the development of 1D core/shell structures such as NWs and NTs appears as a promising solution. These nanoengineered structures offer combined advantages of the two distinct materials, i.e., core and shell. Accordingly, Grote et al.^[^ ^239^ ^]^ synthesized SnO_2_/MnO_2_ core–shell NTs via a unique template‐based fabrication technique offering precise control over several structural parameters, as exemplified in Figure 21j. First, a SnO_2_ core was prepared via ALD, followed by electrodeposition of a thin MnO_2_ shell over the core (Figure 21k). In this configuration, the SnO_2_ core provided a highly conducting matrix, whereas the thin MnO_2_ core entailed a short diffusion length. During the standard CV tests, Mn experiences redox behaviors at the electrode surface (i.e., MnO_2_ shell). The involved reactions are stated as follows:^[^ ^239^ ^]^

(21) $${\mathrm{MnO}}_{2}+M^{+}+e^{-}↔\mathrm{MnOOM}\left(M=H^{+}{\mathrm{or}\mathrm{Na}}^{+}\right)$$

When used as an SC electrode, the prepared SnO_2_/MnO_2_ core–shell nanostructure exhibited superior electrochemical characteristics, i.e., high capacitance (910 F g^–1^ at 1 A g^–1^), moderate rate performance of 217 F g^–1^ at 50 A g^–1^ with excellent cycling stability at 5 A g^–1^ over 2600 cycles (Figure 21l,m).

**Figure 22:**
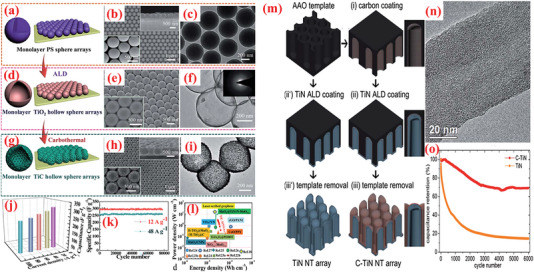
a,d,g) Visual representation of fabrication of monolayer TiC hollow sphere arrays (HSAs) on a graphite paper. b,c) SEM–TEM images of PS sphere template. e) and f) SEM–TEM images of the TiO_2_ HSAs. h) and i) SEM–TEM images of TiC HSAs. j) Specific capacitances at various current densities. k) High‐rate cycle life. l) Ragone plots of TiC HNSA‐based SCs Reproduced with permission.^[^ ^324^ ^]^ Copyright 2016, Royal Society of Chemistry. m) Visual representation of fabrication of self‐supported C–TiN (route A) and TiN (route B) NTAs. Route A: first, a carbon layer is synthesized on the AAO template from self‐assembled PNMs followed by annealing procedure. Then, ALD grown TiN layer on the carbon coated AAO template; route B: ALD‐TiN layer is straight gorwn onto the AAO template. (n) TEM image of a single CNT with a diameter of ≈60 nm. o) Cyclic performance of the C–TiN and TiN NTAs electrodes. Reproduced with permission.^[^ ^325^ ^]^ Copyright 2015, Royal Society of Chemistry.

Interstitial metal carbides with special qualities including high electrical conductivity, high hardness, and strong resistance to oxidation and corrosion are excitingly emerging as a new favorite for the advance electrodes. Xia et al.^[^ ^324^ ^]^ prepared excellent electrode material for high‐temperature (65 °C) organic SCs monolayer composed of titanium carbide (TiC) hollow nanosphere arrays (HSNAs) obtained via a facile ALD‐assisted template methods indicated in **Figure** **22**a–g. Thus, the prepared electrodes offered high capacitance, superior long‐term cyclic stability (maintained 98% over 75 000 CD cycling), and superior rate performance at high current rates (291 F g^–1^ at 12 A g^–1^ and 255 F g^−1^ at 48 A g^–1^). Notably, even at 65 °C, the TiC HNSA electrodes provided high specific capacitances with a retention of 80.7% from 12 to 96 A g^–1^ and could stably operate at up to 3 V without electrolyte degradation in Figure 22j–l.^[^ ^324^ ^]^ To fabricate nanostructured composite materials, a versatile method was adopted in ref. [325] in which the authors combined the well‐established AAO technique with a novel carbon coating approach based on the self‐assembly of polymeric nano micelles (PNMs) onto a AAO template. Briefly, a functional carbon layer was initially deposited inside the pores of the AAO template, followed by the deposition of the active material within it via a facile ALD process. By employing this approach, self‐standing nanostructures of carbon coated TiN (C–TiN) nanotube arrays were developed as advance electrode materials (Figure 22m,n. Applied as a SC electrode, the C–TiN nanotube arrays exhibited capacitances values of 167 and 95 F g^–1^ at 1 and 50 A g^–1^. Comparably, the TiN nanotube arrays‐based electrode delivered 140 and 61 F g^–1^ at 1 and 50 A g^–1^. As observed, >70% of the capacity was retained by the C–TiN nanotube array electrode for over 6000 cycles, which was greater than 4.5 times to that retained by the bare TiN electrode (Figure 22o). This performance disparity between the two electrodes can be explained as an effect of carbon shell in C–TiN electrodes, which acted as a cage for accommodating the cycling‐induced stresses in TiN, thereby preventing the structural breakdown and electrode pulverization.

Existing of the current research work concerning supercapacitor electrodes fabricated/engineered using ALD indicates several key elements such as thicknesses or number of layers, deposition temperature, etc. that significantly influence the capacitive attributes of nanocomposite electrodes. A paramount factor of consideration is the thickness of the ALD coating, which has been observed to directly impact the electrochemical performance of different pseudocapacitive materials. By controlling and adjusting the cycle numbers/layers of the coating for various applications, we can gain insights that could pave the way to creating superior electrode materials with ideal ALD thickness. Additionally, the surface chemistry of the substrate material, particularly its porous/tunnel‐type structure, significantly influences the ALD process. It affects crucial parameters like ALD nucleation, penetration, and layer uniformity. As a result, it influences various aspects of the electrochemical performance. For example, substrates that have pores larger than the ALD precursor tend to be more favorable for effective precursor diffusion, leading to a superior coating. On the other hand, substrates with smaller pores could hinder precursor diffusion and reduce the available surface area for coating, thereby impacting the overall electrochemical performance. Therefore, gaining a thorough comprehension of the pore structure of the substrate electrode, as well as its theoretical specific surface area, becomes essential for achieving optimized performance. The ALD deposition temperature is another significant factor. For example, pseudocapacitive metal oxides perform better in a hydrated state due to enhanced charge/ions capacity, which can be improved through lowering the process temperature of the active films during ALD. When it comes to crystalline phase metal sulfides, deposition at lower temperatures results in a hexagonal phase known to improve electrolyte ion intercalation, thereby boosting the active regions of redox process. In addition to depositing current collectors, ALD has successfully demonstrated the deposition of insulating layers in MSC.^[^ ^131^, ^160^, ^162^, ^184^ ^]^ For instance, Bounor et al.^[^ ^326^ ^]^ demonstrated an interesting parallel plate configuration of 3D MSCs integrated on Si‐wafer using thickness controlled MnO_2_ as active material and 5 m LiNO_3_ as electrolyte. The Pt current collector and Al_2_O_3_ insulating layer were deposited via ALD. The proposed 3D strategy yielded high specific area, rapid electron movement, and swift ion distribution. By fine‐tuning the 3D design and film thickness, the fabricated MSCs exhibited a cell high capacitance (0.75 F cm^–2^) with remarkably high energy density (0.05–0.1 mW h cm^–2^, which was higher than the state‐of‐the‐art MSCs). Furthermore, the MSCs exhibited good cycling performance (≈84% retention after 10,000 cycles) at a high‐power density of >1 mW cm^–2^.^[^ ^326^ ^]^

To sum up, the ALD technique has garnered significant attention in recent years due to its clear superiority over other conventional fabrication methodologies, such as electrochemical deposition, PVD, CVD, and various wet chemical processes like hydrothermal/solvothermal techniques. One of the key reasons for the superiority of ALD lies in its inherent benefits. ALD offers precise control over the film thickness at the atomic level, enabling uniform and conformal coatings even on complex and high‐aspect‐ratio substrates. This level of control ensures the deposition of defect‐free and pinhole‐free films, leading to improved structural integrity and enhanced electrochemical performance of the resulting nanocomposite electrodes. The evaluation of various key parameters in ALD underscores their significance and intricacy in establishing the processing–property relationship of the nanocomposite electrodes. However, due to the limited research available, presenting a single illustrative relationship among all parameters can be challenging. In **Table** **2**, We have meticulously summarized the outcomes of the supercapacitors' performance concerning capacitance value and cyclic stability tests for ALD‐inspired electrodes, and in comparison, to other techniques. The results unequivocally demonstrate the superior capacitance values and enhanced cyclic stability of ALD‐based electrodes, reaffirming their immense potential as a reliable and cutting‐edge technology in the field of energy storage. However, to achieve the best electrochemical performance, it is essential not only to focus on the rational design and synthesis of novel nanostructured electrodes but also to have a profound knowledge of ALD parameters and their proper manipulation. Researchers need to delve deeper into understanding the impact of parameters like precursor choice, temperature, and exposure time on film properties and how they affect the electrochemical behavior of the nanocomposite electrodes. Looking forward, continued investigations and innovative designs of hybrid nanocomposites hold immense potential for the future of ALD research in supercapacitor applications. By exploring new combinations of materials and optimizing ALD parameters, researchers can pave the way for even more efficient and high‐performing supercapacitors, meeting the increasing demands for energy storage in various industries and applications. With these advancements, ALD‐based nanocomposite electrodes are expected to emerge as the frontrunners in the development of next‐generation energy storage devices.

**Table 2:** Comparative analysis of SCs results: ALD‐grown electrodes vs. other preparation methods.

ALD grown electrodes	Supercapacitors performance	Electrodes prepared by other methods				Ref.
		Electrode name	Achieved Capacitance [F g^–1^ A g^–1^]	Cycle stability [times, A g^–1^]	Retention [%]	
**TiO_2_‐Graphene**	Capacitance: 84 F g^–1^ at 10 mV s^–1^; Cycle stability: 1000 cycles at 2 A g^–1^; Retention: 87.5%	3DG/TiO_2_ composite rGO–TiO_2_ NPs	X 62.8/0.125 2	500 cycles/2 X	86 X	[327, 328]
**NiO**	Capacitance: 622 F g^–1^ at 2 A g^–1^; Cycle stability: 4000 cycles at 50 A g^–1^; Retention: 74%	NiO NPs NiO nanocolumns NiO nanoflakes NiO nanoslice	132/5 mVs^−1^ 390/5 410/0.5 mA cm^–2^ 333/2	500 1000/5 500/10 mA cm^–2^ 400	75 X 92 90	[329, 330, 331, 332]
**Fe_2_O_3_**	Capacitance: 787 F g^–1^ at 1 A g^–1^; Cycle stability: 5000 cycles at 50 A g^–1^; Retention: 91.6%	flower‐like Fe_2_O_3_ Fe_3_O_4_@C Fe_3_O_4_ NPs Fe_2_O_3_ Fe3O4 nanocubes	127/1 110.8/0.5 274 71.7 65.4/0.5	1000 2000 5000 1000 3000	80 95.6 83 64 X	[333, 334, 335, 336, 337]
**Co_3_O_4_**	Capacitance: 705 F g^–1^ at 1 A g^–1^; Cycle stability: 5000 cycles; Retention: 94%	D‐nanonet Co_3_O_4_ net‐like Co_3_O_4_ Co_3_O_4_ films	656/ 30 mV s^–1^ 345/2 155/2.75	1000/5 2500/2 1000/2.75	90.2 X 72.2	[338, 339, 340]
**V_2_O_5_**	Capacitance: 1550 F g^–1^ at 1 A g^–1^; Cycle stability: 5000 cycles; Retention: 92%	V_2_O_5_‐nb film yolk–shell V_2_O_5_ V_2_O_5_‐rGo V_2_O_5_ NPs V_2_O_5_ nanorods rGO/V_2_O_5_ V_2_O_5_ nanoporous	132.5/1 704.17/1 289/0.01 310/1 417/0.5 484/0.5 316	1000 4000/3 1000/0.06 X 1000/10 1000/10 600/1	94 89 85 X 76 83 76	[341, 342, 343, 344, 345, 346, 347]
**MoS_2_**	Capacitance: 3400 mF cm^−2^ at 3 mA cm^−2^; Cycle stability: 4500 cycles; Retention: 82%	MoS_2_@CNT/RGO MoS_2_/CoS_2_ MoS_2_ thin films NiO/MoS_2_/rGO	129 mF cm^−2^ at 0.1 mA cm^−2^ 142 mF cm^−2^ at 1 mA cm^−2^ 71 mF cm^−2^ at 1 mV s^−^ 7.4 mF cm^−2^ at 25 mV s^−^	10000 1000 X 1000	94.7 92.7 X 90	[348, 349, 350, 351]
**Co_9_S_8_**	Capacitance: 1300 m F cm^–2^(1800 F g^–1^) at 0.7 mA cm^–2^; Cycle stability: 50 000 cycles; Retention: 98%	GH@NC@Co_9_S_8_ (Co_0.94_Fe0.06)_9_S_8_ Co_9_S_8_/C Co_9_S_8_ NPs	842.4 mF cm^−2^ /1 454/1 756.2/1 718/1	8000 5000 2000 10000	95.8 94 73.4 83.1	[352, 353, 354, 355]
**SnS_x_**	Capacitance: 805.5 m F cm^–2^ at 0.5 mA cm^–2^; Cycle stability: 5000 cycles; Retention: 90%	SL‐SnS_2_ SnS_2_‐gC_3_N4/rGO SnS_2_/SnS SnS_2_/MoS_2_ SnS_2_ nanosheet	117.11/1 403/0.5 350/0.5 105.7/2.35 194.4mF cm^−2^	2000 1500/4 2000 1000 10000	80 63.8 95 90.4 86.5	[356, 357, 358, 359, 360]
**RuO_x_**	Capacitance: 644 F g^–1^; Cycle stability: 10 000 cycles; Retention: 117%	PS/RuO_2_ nanosphere RuO_2_ nanorods RuO_2_ EG@RuO_x_	258 188/1mA cm^–2^ 510/1 205/0.5	500 3000 3000 10000	83.4 93 87 86	[361, 362, 363, 364]
**NiO/Co_3_O_4_**	Capacitance: 2760 F g^–1^ at 2 A g^–1^; Cycle stability: 12 000 cycles; Retention: 95.5%	NiO‐Co_3_O_4_‐rGO NiO@Co3O4 NFs NiO@Co_3_O_4_ HOrGO/TMOs	894/0.5 437/1 1769.2/1 910	1000 1000 10000 2000	91 82.9 87.5 89.9	[365, 366, 367, 368]
**NiCO_2_O_4_/MoO_2_**	Capacitance:1732.5 C g^−1^; Cycle stability: 20 000 cycles; Retention: 90%	MnO_2_@NiCo_2_O_4_ NiCo_2_O_4_@MnO_2_ nanoneedle	3086 mF cm^−2^ at 2 mA cm^−2^ 1001/15	6000 10000	97.3 87.4	[369, 370]
**NiO‐LDH/NiOOH/ALD‐NiO**	Capacitance: 204.5 F g^–1^ at 1 A g^–1^; Cycle stability: 10 000 cycles; Retention: 90.9%	(Ni,Co)Se_2_/NiCo‐LDH//PC NiCo‐LDH@NiOOH	102/2 150.4/0.3	3000 10000	90 77.6	[371, 372]
**SnO_2_/MnO_2_ NTAs**	Capacitance: 910 F g^–1^ at 1 A g^–1^; Cycle stability: 2600 cycles; Retention: X%	MnO_2_@SnO_2_	541.6/1	2500	86	[373]
**C–TiN**	Capacitance: 167 F g^–1^ at 1 A g^–1^; Cycle stability: 6000 cycles; Retention: 70%	TiN/C TiN/C	159/0.5 102.6/1	X 5000	X 92	[374, 375]

## 4 Future Perspective and Summery

### 4.1 Current Challenges and Future Direction

Although ALD is incredibly promising as a state‐of‐the‐art technology for fabricating well‐defined 3D nanoarchitectures with improved performance for SCs, the application of ALD in SCs is still in its nascent phase because of certain challenges:

- As ALD originated in the semiconductor industry, the precursors for depositing MOs and nitrides are commonplace. However, for high‐performance SCs, other materials including layered 2D materials (e.g., TMDCs, MXenes), MOFs, and covalent organic frameworks (COFs) are particularly attractive.^[^ ^225^, ^376^, ^377^, ^378^, ^379^, ^380^, ^381^, ^382^, ^383^, ^384^, ^385^, ^386^, ^387^, ^388^, ^389^, ^390^, ^391^, ^392^ ^]^ Therefore, the design of novel ALD processes for growing new electrode materials has emerged as a research hotspot. Moreover, the doping of electrode materials is a well‐recognized approach to improving the SCs performance. However, the difficulty in precisely controlling the dopant concentration negatively influences the deposition of the doped materials with ALD. Depositing certain metals, and metal nitrides/oxides/sulfides using ALD still poses a significant challenge. Metals like Bi, Pb, Ga, Sn etc. are particularly difficult to deposit using ALD because of their high reduction potential and susceptibility to oxidation. Furthermore, depositing certain higher oxidation state metal nitrides in a reduced and conductive state through ALD also presents a substantial difficulty. In addition, depositing mixed metal oxides and managing the reactivity of metal precursors to achieve high electrochemical performance remains a complex task due to the presence of multiple oxidation states. There's a pressing need for substantial research to explore the understanding of the electrode/electrolyte interface to prevent the degradation of the ALD‐deposited active materials during the electrochemical process.^[^ ^123^, ^125^, ^142^, ^205^, ^393^ ^]^
- In addition to novel electrode materials, the preparation of new precursors has become crucial because of the arrival of novel separators as well as passivating materials. Furthermore, the ALD processes for other metals, halides, sulfides, nitrates, and organometallic reactants are inefficient. Currently, precursors are seldom used in ALD because of their high cost and environmentally harmful nature. Therefore, large‐scale economic production of selectively reactive and safe precursors is imminently required.
- The inherently self‐limiting nature severely impedes the atomic‐scale deposition rate of ALD, which is excessively slow in comparison to conventional chemical and physical film growth methods, e.g., sol–gel, sputtering, PLD, CVD. The sluggish growth in each cycle restricts the fabrication rate and increases its cost.
- The industrial‐scale utilization of ALD for commercially viable SCs necessitates the development of innovative ALD reactors. As an example, the integration of ALDs in industrial production systems should be deployed as a single‐step processing technique with a continuous ALD mechanism *sans* purging and vacuum. Given that ALD is a method characterized by slow nucleation and growth, its adoption in energy industries necessitates the scaling up of this technique.

To address these challenges, noble metals and metal oxides are the most frequently deposited materials via ALD. More recently, certain studies demonstrated the successful deposition of other metallic compounds via ALD (e.g., nitrides, selenides, sulfides, and fluorides). These compounds generally offer exciting properties that differ strongly from those of corresponding MO‐based compounds. For instance, nitrides‐based compounds exhibit improved chemical stability and higher electrical conduction than their oxide counterparts, thereby offering attractive avenues for fabricating high‐conductivity electrodes for SCs. Similarly, metal fluorides/selenides are electrochemically stable across wide ranges of potential gradients, which renders them attractive for high‐voltage SCs. Another potential avenue of exploration involves the creation of hybrid nanostructures that incorporate ALD modifications or protections in conjunction with nanostructured electrodes. To date, researchers have only explored a limited number of active materials through the ALD technique for creating hybrid nanocomposites. As a result, there is a substantial need to identify the optimal combination of nanocomposite materials. While doping is known to improve the SCs performance, the deposition of doped materials via ALD presents a challenge. This is because ALD struggles to control dopant concentrations at lower levels. Therefore, novel doping strategies need to be developed in the future overcoming this problem. Coating with ALD has also emerged as a promising method for achieving higher cell voltages and/or lower impedances. This discovery underscores the value of further exploring the advantages of nano‐enabled carbon electrodes. Consequently, the challenges of enhancing cell voltage and cycle life in supercapacitors are gradually becoming more surmountable. Looking ahead, it is anticipated that a broader range of coating materials will be available for surface modification of SC electrodes via ALD. This could include metal oxides such as HfO_2_, SnO_2_, B_2_O_3_, etc.; metal fluorides like AlF_3_; metal oxyfluorides; and various other compounds. As electrode materials typically vary in their inherent properties, it's expected that the selection of a coating material will need to be specifically tailored to suit a particular electrode.^[^ ^196^, ^205^ ^]^

To date, several innovative modifications have been introduced in the conventional ALD process. For instance, plasma‐enhanced ALD (PEALD) enables the use of low reactivity materials as precursors for depositing highly crystalline thin‐films at relatively low growth temperatures. Another variant, photoassisted ALD provides controlled activation at low reaction temperatures. The high‐temperature ALD deposits 3D nanoarchitectures in highly confined spaces at elevated growth temperatures. Moreover, roll‐to‐roll ALD and thermal ALD processes have been reported to offer high deposition rates along with significant improvements in crystallization. Furthermore, spatial atmospheric ALD (AALD) has been successful in separating the precursors in space instead of time, with growth occurring proximate to the atmospheric pressures. Recently, “particle‐ALD” comprising fluidized particle bed reactor and rotary ALD reactor was proposed for efficient ALD coating over powders/nanoparticles and to circumvent the coverage issues caused by large surface areas and abundant active sites. During the ALD growth, the particles are transported using inert gas flow and rotation of the chamber or the sample container. Although these modifications impart unprecedented improvements in the conventional ALD for applications in SCs, further interpretation of the precursor transport and growth dynamics is required for efficiently leveraging the precursor and achieving improved coating uniformity.

Going forward, we anticipate that ALD will play a pivotal role in advancing the application of SCs within emerging customized electronic markets. Notably, the transportation industry, Internet‐of‐Things, wearable electronics, and portable water purification systems are poised to benefit significantly from the incorporation of lightweight and flexible ALD‐based modules. These modules are capable of providing comparable reliability to conventional, rigid modules encapsulated with glass. This technological advancement has the potential to revolutionize the energy storage landscape, meeting the diverse demands of modern electronic devices and creating more efficient and sustainable solutions for various industries. Additionally, because of cost constraints, the mass production of ALD‐deposited coatings on various materials for electrode synthesis should be demonstrated for large‐scale adoption of this deposition technique. In addition to the cost factor, the deposition time involved in ALD growth is concerning. Owing to the inherent growth mechanism, i.e., atomic‐scale deposition, ALD processes require a long period for material deposition compared to alternative deposition techniques such as sputtering, CVD. In this regard, ALD is more suited for the surface modification of predeposited materials than for the direct growth of the materials via this technique.

Overall, ongoing research should focus on the development of novel materials to optimize the ALD growth parameters such as precursors, deposition temperature, and deposition period. In addition, the identification of suitable precursors for the existing ALD processes require more intense research focus as most precursor materials are expensive, rare, and toxic. Thus, these research directions offer exciting opportunities to further leverage the benefits of ALD in energy‐storage applications. We foresee significant progress in this nascent field as it brings new demands on conventional ALD technologies, including revolutionizing traditional ALD setups for scale‐up production, advancements in ALD‐enhanced electrode materials for SCs, exploration on multifunctional ALD coatings etc. Hence, there is ample room for significant innovations and advancements in this field, providing an opportunity for researchers and industry professionals to make groundbreaking discoveries and gain a deeper understanding of the subject.

### 4.2 Conclusions

ALD is considered a potential and adaptable method for fabricating a wide range of films on multiple substrates with adequate conformity, excellent thickness/size control, and excellent stoichiometry, regardless of complex structures and high aspect ratios. These advantages of ALD are particularly attractive for the development of advanced active materials for batteries and SCs. In this review, we explored the fabrication and electrochemical performance of the ALD‐deposited electrode materials, as employed in SCs. In addition to depositing nanosized particles and thin‐films of novel metals and their composites, ALD has been employed to produce a diverse array of electrode structures such as 3D hollow nanostructures in combination with standard synthesis techniques. As such, these nanoarchitectures remarkably enhanced the active surface area and shortened the pathways of charge diffusion, which considerably improved the performance of the as‐fabricated devices. Finally, we suggested potential future research directions to completely leverage the merits of ALD for developing future‐generation SCs.

## Conflict of Interest

The authors declare no conflict of interest.
